# Comparative cardiovascular outcomes and safety of hypoglycemic drug classes in patients with type 2 diabetes and hypertension: a multicenter cohort analysis

**DOI:** 10.1186/s12933-025-02892-5

**Published:** 2025-08-20

**Authors:** Zhiyuan Wei, Wanqian Xu, Yu Wang, Yu Tian, Zhongmin Wang, Shenqi Jing, Weina Liu, Sipeng Shen, Chenlong Qin, Xin Zhang, Jingsong Li, Yun Liu

**Affiliations:** 1 Department of Medical Informatics, School of Biomedical Engineering and Informatics, Nanjing Medical University, Nanjing, 211166 Jiangsu China; 2 Institute of Medical Informatics and Management, Nanjing Medical University, Nanjing, 210029 Jiangsu China; 3 General Practice Medical Center, Chinese Evidence-Based Medicine Center, West China Hospital, Sichuan University, Chengdu, 610041 Sichuan China; 4 Research Center for Scientific Data Hub , Zhejiang Lab , Hangzhou, 311100 Zhejiang China; 5 Engineering Research Center of EMR and Intelligent Expert System, Ministry of Education, College of Biomedical Engineering and Instrument Science, Zhejiang University , Hangzhou, 310027 Zhejiang China; 6 Medical Technology Administration Office, The First Affiliated Hospital with Nanjing Medical University , Nanjing, 210029 Jiangsu China; 7 Department of Information , The First Affiliated Hospital with Nanjing Medical University , Nanjing, 210029 Jiangsu China; 8 Department of Nutrition and Food Hygiene, School of Public Health , Southeast University , Nanjing, 210009 Jiangsu China; 9 Department of Biostatistics, Center for Global Health, School of Public Health, Nanjing Medical University, Nanjing, 211166 Jiangsu China; 10 Department of Geriatrics, Division of Geriatric Endocrinology , The First Affiliated Hospital with Nanjing Medical University , Nanjing, 210029 Jiangsu China

**Keywords:** Type 2 diabetes, Hypertension, Major adverse cardiovascular events (MACE), Cardiovascular safety, Real-world evidence, Retrospective study

## Abstract

**Background:**

Patients with type 2 diabetes (T2D) and hypertension are at increased risk of adverse cardiovascular (CV) events. However, real-world evidence comparing the CV effectiveness and safety of major hypoglycemic drug classes remains limited in this population. This multicenter pooled analysis aims to directly compare the CV outcomes and safety profiles of these key agents in patients with T2D and hypertension.

**Methods:**

We analyzed electronic health records from two databases in a cohort study of T2D patients with hypertension who had initiated metformin as first-line therapy. Propensity score matching (PSM) and Cox proportional hazards models were used to compare the risks of 3-/4-point major adverse cardiovascular events (MACE) and safety outcomes across drug classes added to metformin: insulin, sulfonylureas (SUs), glucagon-like peptide-1 receptor agonists (GLP-1 RAs), dipeptidyl peptidase-4 inhibitors (DPP4is), glinides, acarbose, and sodium-glucose transporter 2 inhibitors (SGLT2is).

**Results:**

Compared with insulin, GLP-1 RAs, DPP4is, and glinides were associated with a lower risk of 3-point MACE (HR: 0.48 [0.31–0.76], 0.70 [0.57–0.85], and 0.70 [0.52–0.94], respectively). SUs were associated with a higher risk of 3-point MACE compared with DPP4is (HR: 1.30 [1.06–1.59]). DPP4is, GLP-1 RAs, and glinides showed a lower risk of 3-point MACE compared with acarbose (HR: 0.62 [0.51–0.76], 0.47 [0.29–0.75], and 0.59 [0.43–0.81], respectively). Similar patterns were observed for 4-point MACE. For safety outcomes, DPP4is were associated with a reduced risk of chronic kidney disease, while insulin use was associated with reduced risks of inflammatory polyarthritis and insomnia. However, DPP4is were associated with higher risks of coronary atherosclerotic diseases and hypertensive heart disease.

**Conclusions:**

This study highlights the differential cardiovascular effectiveness and safety profiles of hypoglycemic therapies in real-world settings, providing valuable insights for optimizing T2D management, particularly in patients with comorbid hypertension.

**Supplementary Information:**

The online version contains supplementary material available at 10.1186/s12933-025-02892-5.

## Research insights

**What is currently known about this topic?**

Limited real-world evidence on CV safety of hypoglycemic agents in T2D+hypertension.

**What is the key research question?**

Compare novel vs. traditional agents on MACE/safety in T2D+hypertension.

**What is new?**

GLP-1 RAs/DPP4is show lower MACE vs. insulin/acarbose; DPP4is reduce CKD risk.

**How might this study influence clinical practice?**

Prioritize GLP-1 RAs/DPP4is for T2D+hypertension to improve CV outcomes.

## Introduction

Over the past decade, there have been substantial changes in the therapeutic approaches for type 2 diabetes (T2D). Novel hypoglycemic agents, targeting sodium-glucose transporter 2 and glucagon-like peptide-1 receptors, have emerged, offering dual benefits of reducing blood glucose levels and directly lowering cardiovascular risk in patients [1]. Considering the high incidence of cardiovascular events in individuals with hypertension [2], novel hypoglycemic drugs represent a new therapeutic option for patients with concurrent T2D and hypertension.

A series of clinical trials designed to evaluate the cardiovascular safety of sodium-glucose transporter 2 inhibitors (SGLT2is) and glucagon-like peptide-1 receptor agonists (GLP-1 RAs) found that these medications have been shown to reduce major adverse cardiovascular events (MACE), including myocardial infarction (MI), hospitalization for heart failure (HF), and cardiovascular mortality [3]. However, for dipeptidyl peptidase-4 inhibitors (DPP4is), sulfonylureas (SUs), glinides, acarbose, and insulin, which were widely used prior to the introduction of these newer drugs, no similar trials have been conducted to evaluate their cardiovascular efficacy or safety [4]. Nevertheless [3], these drugs continue to be extensively utilized in clinical practice within certain countries and regions, and are recommended as second-line pharmacotherapy for T2D in clinical practice guidelines [5].

To identify existing gaps and limitations in the current literature, we conducted a systematic review. After carefully applying inclusion and exclusion criteria, we identified 16 relevant studies (Supplementary Tables, Table S6), including 11 observational or retrospective studies and 5 systematic reviews and meta-analyses of randomized controlled trials (RCTs). These studies primarily evaluated cardiovascular efficacy and safety outcomes for novel hypoglycemic agents compared with placebo or standard care. However, several important gaps remain in the current evidence. First, existing RCTs have primarily assessed new hypoglycemic agents as add-on therapies rather than comparing them directly with traditional medications, resulting in uncertainties regarding their comparative cardiovascular effectiveness and safety [6, 7]. Second, there is still a notable lack of comprehensive, large-scale head-to-head direct comparisons among novel agents [8]. Finally, current evidence regarding cardiovascular and safety profiles across diverse patient populations is still insufficient, particularly for individuals with both T2D and hypertension [9]. These evidence gaps hinder the development of precise and individualized therapeutic recommendations for this patient group.

Although the 2024 American Diabetes Association (ADA) Standards of Care in Diabetes have explicitly recognized hypertension as a significant risk factor for the occurrence and progression of chronic kidney disease [10], formulating therapeutic recommendations for patients with T2D and concomitant hypertension on the basis of existing clinical evidence still poses several challenges [11].

Inspired by the LEGEND-T2DM (Large-Scale Evidence Generation and Evaluation Across a Network of Databases for Type 2 Diabetes Mellitus) initiative [12], we conducted a study to compare common hypoglycemic drug treatments in patients with T2D and concomitant hypertension by employing a multicenter cohort analysis across observational databases from the Observational Health Data Science and Informatics (OHDSI) distributed data network, which utilizes data from multiple centers and performs integrated comparisons via analytical techniques to mitigate confounding factors [13]. Our study, carefully constructed by experts using a previously validated methodological framework and drawing on existing evidence to minimize bias and improve reproducibility, offers a holistic perspective on the results and their consistency across diverse populations, medications, and outcomes. We report results from comparisons of combination therapies involving two drugs, using data from participating sources through June 2023, covering patients from July 1996 to March 2023.

## Research design and methods

### Data source

In this cohort study, we analyzed patient records from two Electronic Health Record (EHR) databases mapped to the Observational Medical Outcome Partnership (OMOP) Common Data Model (CDM) version 5.3. This is the first large-scale analytical study involving centers exclusively from mainland China. The databases, provided by research partners within the OHDSI community, include Jiangsu Provincial People's Hospital (JSPH) and the First Affiliated Hospital, Zhejiang University School of Medicine (FAHZU), containing records of 202,146 and 332,328 patients, respectively.

We employed the OHDSI federated network model, performing deidentified data access and statistical analyses within each institution using the OHDSI common tool stack. The analytical process was predefined, and consolidated results were gathered for interpretation.

### Study population

The study population included individuals with T2D and hypertension who had previously received metformin monotherapy and initiated an upgraded treatment with one of seven major medications—glinides, SUs, acarbose, insulin, SGLT2is, DPP4is, or GLP-1 RAs—as per diabetes practice guidelines from 2012 to 2024. Patients initiating other drug classes were excluded. Notably, although both SUs and glinides enhance insulin secretion via pancreatic β-cell K_ATP_ channels, they are pharmacologically distinct classes. SUs provide sustained glycemic control, while glinides act rapidly to target postprandial glucose with short duration. Major guidelines (ADA/EASD) and evidence distinguish their indications and safety profiles [14, 15]. Thus, we analyzed them as separate comparator groups. We established seven nonoverlapping exposure cohorts, each comprising patients who escalated from metformin monotherapy to dual combination therapy with one of the seven drug classes. Using a retrospective, comparative new-user cohort design [16], we included patients aged 18 years or older with documented T2D and hypertension diagnoses prior to initiating a second-line agent. Patients were required to have at least 30 days of prior database observation, evidence of metformin use, and no prior exposure to comparator second-line or other antihyperglycemic agents. The new-user cohort design minimizes confounding factors by focusing on patients starting a new drug at follow-up initiation, making it ideal for comparative cardiovascular effectiveness and drug safety studies.

### Outcome measurements

This study assessed 17 outcomes, including effectiveness and safety measures. Effectiveness outcomes included: (1) 3-point MACE comprising acute MI, stroke, and sudden cardiac death (SCD); (2) 4-point MACE, adding hospitalization for HF; and (3) individual MACE components. Outcome cohorts were based on validated phenotypes from prior studies [17, 18]. Safety outcomes covered 10 prevalent conditions in Chinese patients with T2D comorbid with hypertension, such as coronary atherosclerotic disease, chronic kidney disease, inflammatory polyarthritis, hypertensive heart disease without congestive HF, hyperuricemia, osteoporosis, insomnia, urinary tract infections, hepatic failure, and affective disorders. Among these, 3-point MACE and 4-point MACE were the primary outcomes of interest. The remaining outcomes were considered secondary endpoints to provide a broader effectiveness and safety profile. All outcomes were derived using published phenotypes, typically defined by diagnostic codes from inpatient or outpatient records. A consistent, systematic approach was applied to all outcomes. Patients with prior events were identified, and outcomes were constructed using validated phenotypes based on clinical diagnosis codes.

### Statistical analysis

To address confounding factors and enhance cohort balance, we developed propensity score models via logistic regression for each drug class pair, incorporating demographic, clinical, and medical history covariates. Variable-ratio propensity score matching (PSM) was used as the primary analytical method to address confounding factors and achieve cohort balance, using a nearest neighbor algorithm with a caliper of 0.02 standard deviations (SDs) [19]. Inverse probability of treatment weighting (IPTW) was additionally performed as a sensitivity analysis to evaluate the robustness of the findings [20]. Covariate balance was assessed using standardized mean differences (SMDs < 0.1) indicating negligible imbalance [21].

Kaplan–Meier curves visualized cumulative incidence proportions, with log-rank tests evaluating differences. Cox proportional hazards models estimated hazard ratios (HRs) and 95% confidence intervals (95% CIs), adjusted for residual imbalances (SMDs > 0.1) [22]. Pooled analysis estimates were derived using a random-effects model [23].

A total of 1071 effect estimates were generated for seven drug classes (21 pairwise comparisons) across 17 outcomes and two databases, using two propensity adjustment approaches. Comprehensive diagnostics included preference score distributions, cohort balance assessments (pre-/post-adjustment), Kaplan–Meier plots for HR proportionality, and HR estimates. Analyses were conducted in R version 4.2.1, using two-tailed tests (statistical significance: *P* < 0.05) and Benjamini–Hochberg false discovery rate (FDR) correction for multiple comparisons [24].

## Results

### Cohort characteristics

A total of 10,507 patients’ EHRs from two centers (JSPH and FAHZU) were included in the study. Patients received one of seven second-line combination therapies: metformin combined with SUs, insulin, DPP4is, acarbose, SGLT2is, glinides, or GLP-1 RAs. In JSPH, the SUs group had the largest number of patients (n = 745), while the GLP-1 RAs group had the fewest (n = 252). A similar distribution was observed in FAHZU, where the SUs group remained the largest (n = 1609) and the GLP-1 RAs group the smallest (n = 205). The detailed patient counts for all treatment groups in both centers are provided in Supplementary Tables, Table S1.

Median follow-up time differed significantly between treatment groups and centers. Average follow-up time ranged from 17 days (SGLT2is group at FAHZU) to 581 days (DPP4is group at JSPH).

**Fig. 1:**
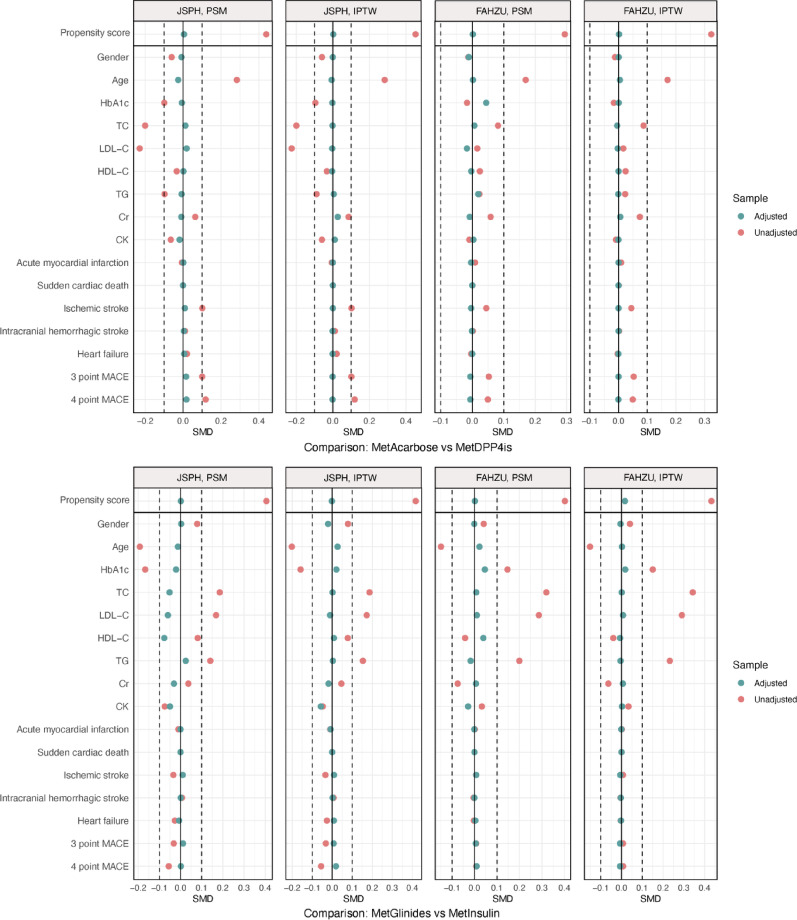
Demographic characteristics for hypoglycemic drug comparison before and after adjustment using PSM and IPTW methods across JSPH and FAHZU databases. This figure presents the comparisons of MetAcarbose (metformin combined with acarbose) versus MetDPP4is (metformin combined with dipeptidyl peptidase-4 inhibitors) in the upper panel and MetGlinides (metformin combined with glinides) versus MetInsulin (metformin combined with insulin) in the lower panel, with results from both PSM and IPTW methods across JSPH and FAHZU databases. Group differences were assessed using SMD, with an SMD value of <  ± 0.1 considered indicative of balance. An SMD > 0 indicates that the characteristic is higher in the target drug class (first), while an SMD < 0 indicates that it is higher in the comparator drug class (second)

The baseline characteristics of the original data from the two databases are summarized in Supplementary Tables (Table S1). The distribution of propensity scores before adjustment is shown in Supplementary Figures (pp. 1–2). After propensity score adjustment (PSM and IPTW), prespecified covariate balance in baseline characteristics was achieved for most comparisons across both databases (Fig. 1 and Supplementary Figures, pp. 3–17). However, for certain comparison groups, specific factors remained imbalanced after adjustment and were further adjusted in the Cox regression model.

Figure 1 presents the comparison of patients initiating acarbose (target) with those initiating DPP4is (comparator) in the JSPH database. Before PSM adjustment, the acarbose group was older, had higher TC and LDL-C levels, and exhibited a higher prevalence of 3-point and 4-point MACE compared to the DPP4is group. However, after PSM adjustment, the acarbose and DPP4is groups were well balanced across all demographic and clinical characteristics. A similar pattern was observed after applying the IPTW method, thereby ensuring the robustness and consistency of the results. In the FAHZU database, before PSM adjustment, patients initiating acarbose were older than those initiating DPP4is, and a similar pattern was observed after applying the IPTW method.

In the comparison of patients initiating glinides (target) with those initiating insulin (comparator) in the JSPH database, before PSM adjustment, the glinides group was younger, had higher TC, LDL-C, and TG levels, and had a lower HbA1c level compared to the insulin group. In contrast, after PSM adjustment, the glinides and insulin populations were well balanced on all demographic and clinical patient characteristics. In the FAHZU database, before PSM adjustment, patients initiating glinides were younger, had higher HbA1c, TC, LDL-C, and TG levels relative to those initiating insulin. After PSM adjustment, the glinides and insulin groups were well balanced. A comparable pattern was observed following the application of the IPTW method.

### Comparative effectiveness endpoints

Our pooled analysis presents comparative effect estimates for seven key cardiovascular events on the basis of PSM across two databases (JSPH and FAHZU).

**Fig. 2:**
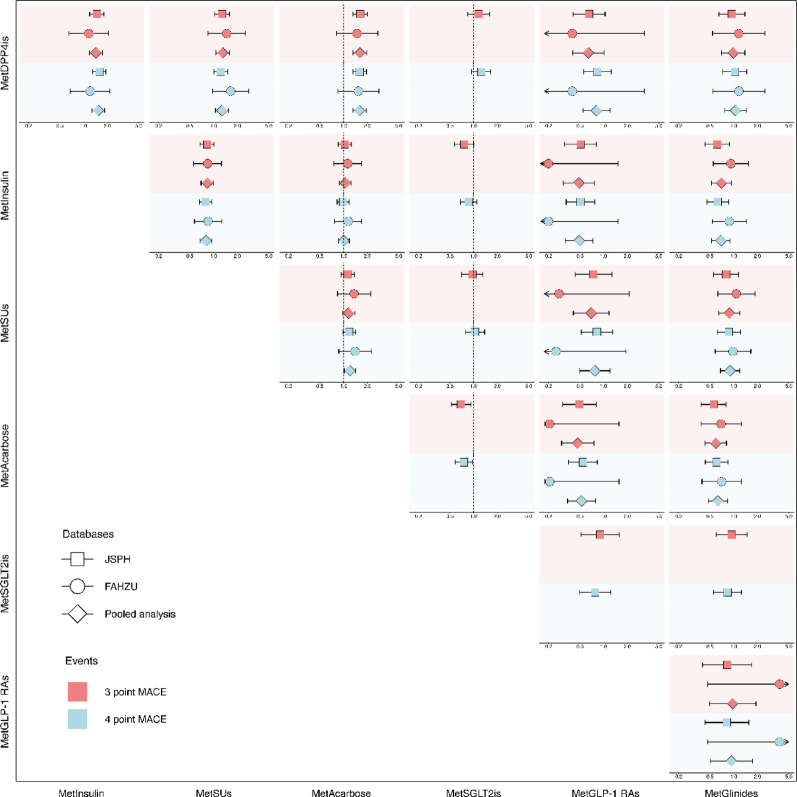
Comparative effectiveness of seven hypoglycemic drug classes on 3-point and 4-point MACE using PSM method in JSPH, FAHZU, and Pooled analysis. Seven hypoglycemic drug classes (MetInsulin [metformin combined with insulin], MetSUs [metformin combined with sulfonylureas], MetAcarbose [metformin combined with acarbose], MetSGLT2is [metformin combined with sodium-glucose cotransporter-2 inhibitors], MetGLP-1 RAs [metformin combined with glucagon-like peptide-1 receptor agonists], MetGlinides [metformin combined with glinides], and MetDPP4is [metformin combined with dipeptidyl peptidase-4 inhibitors]) were compared across JSPH and FAHZU databases, with results combined through pooled analysis. Squares represent results from JSPH, circles from FAHZU, and diamonds from pooled analysis. Red and blue shaded areas indicate results for 3-point MACE and 4-point MACE, respectively. For each drug comparison, the target drug class is in the row, while the comparator drug class is in the column. Points report HR estimates, with lines marking their 95% CIs. An HR > 1 indicates that the risk is higher in the target drug class, while an HR < 1 indicates that it is higher in the comparator drug class. Comparisons were considered statistically significant if the 95% CI did not overlap 1

Table 1 and Fig. 2 summarize the HRs for the above-mentioned seven combination therapies. For 3-point MACE, GLP-1 RAs, DPP4is, and glinides were associated with a lower risk compared with insulin (HR: 0.48 [95% CI 0.31–0.76], 0.70 [95% CI: 0.57–0.85], and 0.70 [95% CI 0.52–0.94]). Compared with DPP4is, SUs were associated with a higher risk (HR: 1.30 [95% CI 1.06–1.59]). Relative to acarbose, DPP4is, GLP-1 RAs, and glinides were associated with a lower risk (HR: 0.62 [95% CI 0.51–0.76], 0.47 [95% CI 0.29–0.75], and 0.59 [95% CI 0.43–0.81]). The 4-point MACE results were consistent with those of 3-point MACE, except for an additional significant finding that SUs were associated with a lower risk compared to insulin (HR: 0.80 [95% CI 0.68–0.95]). The pooled analysis across both centers demonstrated consistent findings, with relatively low heterogeneity. The pooled analysis results for the overall 3-point MACE, 4-point MACE, and individual components of MACE are presented in Supplementary Figures (pp. 28) and Supplementary Tables (Table S2).

**Table 1:** Pooled analysis results of HRs for 3-point and 4-point MACE across hypoglycemic drug classes

Target	Comparator	Events	HR	95% CI	*P-*_*RAW*_* value*	*P-*_*FDR*_* value*
MetInsulin	MetDPP4is	3-point MACE	1.43	1.17–1.74	** < 0.001**	**0.004**
MetInsulin	MetDPP4is	4-point MACE	1.54	1.28–1.85	** < 0.001**	** < 0.001**
MetSUs	MetDPP4is	3-point MACE	1.30	1.06–1.59	**0.01**	**0.028**
MetSUs	MetDPP4is	4-point MACE	1.28	1.06–1.55	**0.012**	**0.030**
MetSUs	MetInsulin	3-point MACE	0.83	0.69–0.99	**0.043**	0.091
MetSUs	MetInsulin	4-point MACE	0.80	0.68–0.95	**0.01**	**0.028**
MetAcarbose	MetDPP4is	3-point MACE	1.61	1.31–1.96	** < 0.001**	** < 0.001**
MetAcarbose	MetDPP4is	4-point MACE	1.59	1.32–1.92	** < 0.001**	** < 0.001**
MetAcarbose	MetInsulin	3-point MACE	1.05	0.88–1.25	0.597	0.695
MetAcarbose	MetInsulin	4-point MACE	1.00	0.85–1.18	0.96	0.960
MetAcarbose	MetSUs	3-point MACE	1.16	0.96–1.39	0.122	0.216
MetAcarbose	MetSUs	4-point MACE	1.20	1.01–1.43	**0.035**	0.078
MetGLP-1 RAs	MetDPP4is	3-point MACE	0.64	0.41–1.01	0.054	0.110
MetGLP-1 RAs	MetDPP4Iis	4-point MACE	0.81	0.54–1.20	0.293	0.418
MetGLP-1 RAs	MetInsulin	3-point MACE	0.48	0.31–0.76	**0.002**	**0.009**
MetGLP-1 RAs	MetInsulin	4-point MACE	0.49	0.32–0.73	** < 0.001**	**0.004**
MetGLP-1 RAs	MetSUs	3-point MACE	0.69	0.41–1.17	0.169	0.283
MetGLP-1 RAs	MetSUs	4-point MACE	0.77	0.50–1.21	0.262	0.382
MetGLP-1 RAs	MetAcarbose	3-point MACE	0.47	0.29–0.75	**0.002**	**0.009**
MetGLP-1 RAs	MetAcarbose	4-point MACE	0.52	0.35–0.79	**0.002**	**0.009**
MetGlinides	MetDPP4is	3-point MACE	0.99	0.70–1.39	0.942	0.959
MetGlinides	MetDPP4is	4-point MACE	1.06	0.77–1.45	0.738	0.825
MetGlinides	MetInsulin	3-point MACE	0.70	0.52–0.94	**0.016**	**0.038**
MetGlinides	MetInsulin	4-point MACE	0.69	0.53–0.90	**0.007**	**0.020**
MetGlinides	MetSUs	3-point MACE	0.88	0.65–1.20	0.416	0.551
MetGlinides	MetSUs	4-point MACE	0.90	0.68–1.19	0.459	0.585
MetGlinides	MetAcarbose	3-point MACE	0.59	0.43–0.81	** < 0.001**	**0.006**
MetGlinides	MetAcarbose	4-point MACE	0.63	0.47–0.84	**0.002**	**0.009**
MetGlinides	MetGLP-1 RAs	3-point MACE	0.97	0.49–1.92	0.933	0.959
MetGlinides	MetGLP-1 RAs	4-point MACE	0.93	0.51–1.72	0.826	0.888

The pooled analysis combines data from JSPH and FAHZU databases when both are available. Empty cells indicate results based solely on JSPH database. HRs are presented with their 95% confidence intervals (CIs). An HR > 1 indicates a higher risk in the target drug class, while an HR < 1 indicates a higher risk in the comparator drug class. Comparisons were considered statistically significant if the P value is less than 0.05

In the analysis of heart failure, SUs and acarbose were associated with a significantly lower risk (HR: 0.59 [95% CI 0.41–0.85] and 0.62 [95% CI 0.44–0.88]) compared to insulin.

For ischemic stroke, DPP4is, GLP-1 RAs, and glinides were associated with a lower risk (HR: 0.71 [95% CI 0.58–0.87], 0.50 [95% CI 0.32–0.80], and 0.62 [95% CI 0.45–0.84]) compared to insulin. Compared with DPP4is, SUs were associated with a higher risk of ischemic stroke (HR: 1.29 [95% CI 1.05–1.59]). Compared with acarbose, DPP4is, GLP-1 RAs, and glinides were associated with a lower risk of ischemic stroke (HR: 0.62 [95% CI 0.51–0.76], 0.50 [95% CI 0.31–0.81], and 0.53 [95% CI 0.38–0.73], respectively).

In Supplementary Figures pp. 17–27, we present a total of 21 pairwise comparisons of survival curves for 3-point and 4-point MACE among the 7 drugs. To further validate our findings, we conducted a sensitivity analysis using IPTW (Supplementary Tables, Table S4). The results of this analysis were largely consistent with those of the main analysis shown in Supplementary Figures pp. 30–32, thereby reinforcing the overall consistency and robustness of our conclusions.

### Comparative safety endpoints

Our pooled analysis presents comparative safety estimates for 10 events with high prevalence among patients with T2D comorbid with hypertension on the basis of PSM across two databases (JSPH and FAHZU). Figure 2 and Supplementary Tables (Table S3) summarize the HRs for the above-mentioned seven combination therapies.

**Fig. 3:**
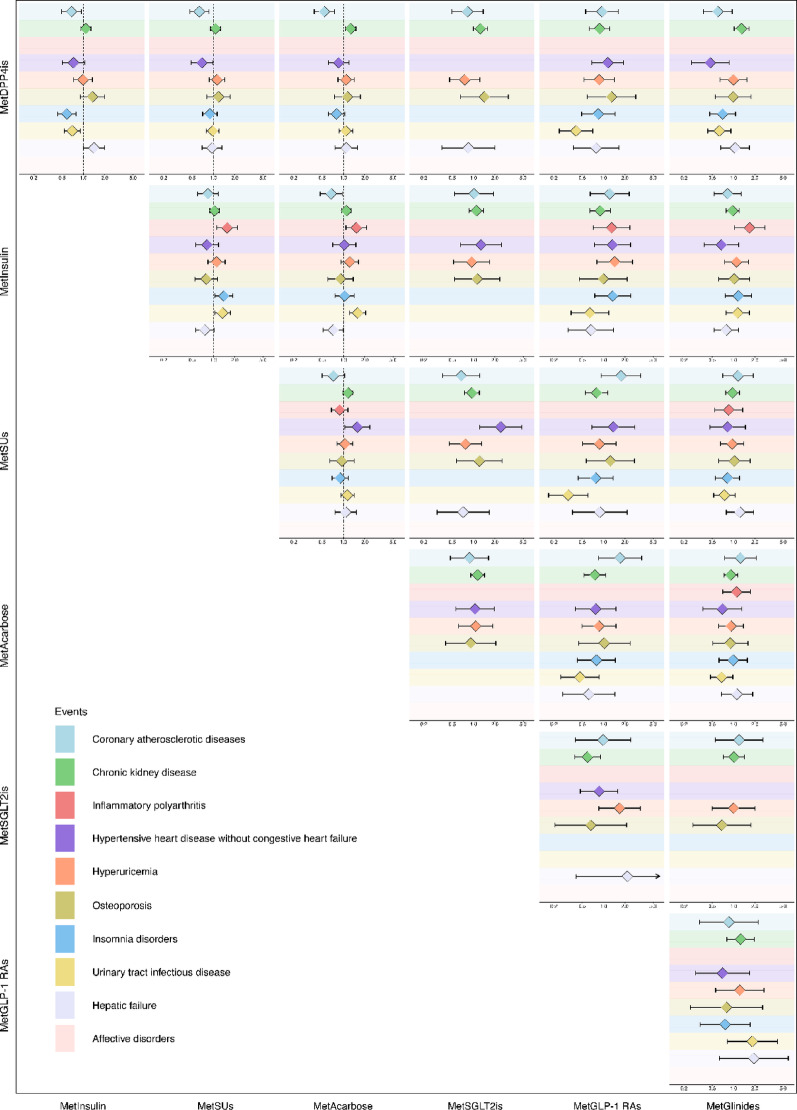
Comparative safety profiles of seven hypoglycemic drug classes across ten outcomes using PSM method in JSPH, FAHZU, and Pooled analysis. Seven hypoglycemic drug classes (MetInsulin [metformin combined with insulin], MetSUs [metformin combined with sulfonylureas], MetAcarbose [metformin combined with acarbose], MetSGLT2is [metformin combined with sodium-glucose cotransporter-2 inhibitors], MetGLP-1 RAs [metformin combined with glucagon-like peptide-1 receptor agonists], MetGlinides [metformin combined with glinides], and MetDPP4is [metformin combined with dipeptidyl peptidase-4 inhibitors]) were compared across JSPH and FAHZU databases, with results combined through pooled analysis. Squares represent results from JSPH, circles from FAHZU, and diamonds from pooled analysis. Different colored bands represent the ten outcomes. For each drug comparison, the target drug class is in the row, while the comparator drug class is in the column. Points report HR estimates, with lines marking their 95% CIs. An HR > 1 indicates that the risk is higher in the target drug class, while an HR < 1 indicates that it is higher in the comparator drug class. Comparisons were considered statistically significant if the 95% CI did not overlap 1

In the analysis of coronary atherosclerotic diseases, insulin, SUs, acarbose, and glinides were associated with a significantly lower risk (HR: 0.68 [95% CI 0.50–0.93], 0.63 [95% CI 0.46–0.86], 0.54 [95% CI 0.39–0.75], and 0.60 [95% CI 0.38–0.96]) compared to DPP4is. Compared with insulin, acarbose was associated with a lower risk (HR: 0.67 [95% CI 0.47–0.96]) (Figure 3).

For chronic kidney disease, acarbose and glinides were associated with a higher risk (HR: 1.27 [95% CI 1.07–1.49], and 1.30 [95% CI 1.02–1.64]) compared to DPP4is.

In the analysis of inflammatory polyarthritis, SUs, acarbose, and glinides were associated with a higher risk (HR: 1.56 [95% CI 1.11–2.18], 1.52 [95% CI 1.09–2.13], and 1.68 [95% CI 1.03–2.75]) compared to insulin.

Compared with DPP4is, both SUs (HR: 0.69 [95% CI 0.48–0.98]) and glinides (HR: 0.47 [95% CI 0.25–0.87]) were associated with a lower risk of hypertensive heart disease without congestive heart failure. The acarbose was associated with a significantly higher risk of hypertensive heart disease without congestive heart failure (HR: 1.57 [95% CI 1.05–2.35]) compared to SUs.

The DPP4is and SUs were associated with a significantly higher risk of insomnia disorders (HR: 1.72 [95% CI 1.27–2.33] and 1.41 [95% CI 1.06–1.87]) compared to insulin.

For urinary tract infectious disease, compared with acarbose, the HRs for GLP-1 RAs and glinides were 0.47 (95% CI 0.25–0.87) and 0.68 (95% CI 0.47–0.98), respectively. In comparison to DPP4is, insulin, GLP-1 RAs, and glinides were associated with a lower risk (HR: 0.70 [95% CI 0.54–0.90], 0.41 [95% CI 0.24–0.71], and 0.63 [95% CI 0.43–0.92]). SUs and acarbose were associated with a significantly higher risk (HR: 1.35 [95% CI 1.04–1.74] and 1.58 [95% CI 1.22–2.06]) compared to insulin. In comparison to SUs, GLP-1 RAs were associated with a lower risk (HR: 0.32 [95% CI 0.17–0.60]).

In the analysis of hepatic failure, DPP4is and acarbose were associated with a lower risk (HR: 0.71 [95% CI 0.51–1.00] and 0.72 [95% CI 0.52–1.00]) compared to insulin.

We present a sensitivity analysis using IPTW, as shown in Supplementary Figures (pp. 33–35) and Supplementary Tables S5. The results of this analysis reinforce the consistency and robustness of our conclusions.

## Discussion

The coexistence of T2D and hypertension confers a markedly elevated risk of MACE, including MI, stroke, and HF. Consequently, comprehensive management of both glycemic and blood pressure control is imperative in this high-risk population. Recent international guidelines, such as the 2024 European Society of Cardiology/European Association for the Study of Diabetes (ESC/EASD) recommendations, underscore the importance of selecting antihyperglycemic agents with demonstrated pleiotropic cardiovascular benefits for individuals with T2D and concomitant hypertension. While previous investigations have largely evaluated cardiovascular outcomes in broader T2D cohorts, our study specifically focuses on patients with both T2D and hypertension—a subgroup in whom optimal cardiovascular risk reduction is especially critical.

To the best of our knowledge, this is the first study independently initiated and led by China within the OHDSI community, aiming to provide robust evidence on the cardiovascular efficacy and safety of hypoglycemic drugs in this unique patient population. We analyzed a large real-world cohort of 10,507 patients from two hospitals in China who, after receiving metformin monotherapy, escalated to dual therapy by adding one of seven major antihyperglycemic drug classes. All pairwise comparisons among the seven second-line agents were performed across 17 health outcomes, providing comprehensive insights into their comparative cardiovascular effectiveness and safety in patients with T2D and comorbid hypertension. The majority of efficacy comparisons revealed no significant differences between the drug classes.

Notably, we found that patients who initiated DPP4is had significantly lower risks for 3-point MACE and 4-point MACE compared to new users of several other dual therapies, including metformin combined with insulin, acarbose, or SUs. However, other drug combinations, such as those GLP-1 RAs or glinides group, showed no statistically significant difference in MACE risk compared to DPP4is group. Similar findings were corroborated in the CAROLINA (Cardiovascular Outcome Study of Linagliptin Versus Glimepiride in Patients with T2D) trial, further substantiating the results of the present study [25]. These findings suggest that DPP4i may be a preferred choice for combination therapy with metformin, offering cardiovascular benefits compared to insulin, SUs and acarbose, while exhibiting comparable efficacy to GLP-1 RAs and glinides.

GLP-1 RAs, a key focus of our study, also performed well in terms of efficacy. Although previous studies have demonstrated the cardiovascular benefits of GLP-1 RAs [1, 3], our real-world comparison between GLP-1 RAs and DPP4is in patients with comorbid T2D and hypertension did not reveal a statistically significant difference in the primary cardiovascular outcomes. This is attributable to the distinct characteristics of this high-risk subgroup and the relatively limited sample size of GLP-1 RAs users in our cohort. Our findings are consistent with the body of evidence from cardiovascular outcome trials, where these agents were evaluated in comparison to placebo [26].

The limited sample size of the SGLT2is group at FAHZU reflects a form of clinical inertia. Diabetologists have identified this clinical inertia as a major barrier preventing patients who meet guideline-based eligibility criteria from receiving optimal SGLT2i therapy [27]. In the analysis from JSPH, SGLT2is were associated with a reduced risk of 3-point MACE (HR: 0.69 [95% CI 0.52–0.92]) and 4-point MACE (HR: 0.75 [95% CI 0.58–0.97]) compared to acarbose, with this benefit being particularly pronounced in reducing the risk of ischemic stroke. However, no significant advantage was observed when SGLT2is were compared with other agents, warranting further investigation.

SUs were associated with a reduced risk of both 3-point and 4-point MACE compared to insulin, with the difference in 4-point MACE remaining statistically significant after FDR correction. Additionally, acarbose showed a trend toward an increased risk of 4-point MACE when compared to SUs-based dual therapy. However, this difference did not reach statistical significance after FDR correction. Class-level comparisons and assessments across large and diverse populations likely contribute to these observed differences. Moreover, these findings do not suggest an absolute benefit from SUs but might even reflect a relative harm from acarbose, as suggested in prior studies [28–32].

Currently, there are few ongoing or planned trials addressing this knowledge gap. Furthermore, a limited number of observational studies have employed singular data sources to assess the cardiovascular outcomes associated with the aforementioned therapeutic agents [33]. The associations observed in the current study, particularly those involving DPP4is, differ from the findings of other studies that used smaller sample sizes. These studies might have been compelled to directly compare drug combinations against SUs due to their limited study population. However, this approach could have introduced challenges in isolating the specific effects of individual drugs within the combinations [34].

In terms of safety, the study findings reveal that DPP4is are at a comprehensive disadvantage in the prevention of atherosclerotic cardiovascular events when compared head-to-head with various hypoglycemic agents. This conclusion aligns with the results of previous real-world studies [35]. Research on the role of DPP4is in atherosclerotic cardiovascular events is limited. Based on the available evidence, we can currently only suggest that DPP4is may not be the preferred choice for the prevention of atherosclerotic cardiovascular events. However, compared to acarbose and glinides, DPP4is demonstrated superior performance in reducing the risk of chronic kidney disease. Our findings provide further validation, from a different perspective, of the SAVOR-TIMI 53 trial—the largest and longest-term efficacy study to date investigating the renal effects of DPP4is [36]. Patients who initiated insulin therapy exhibited reduced risks of inflammatory polyarthritis. Studies have demonstrated that the use of insulin can prevent abnormalities in immune cell regulation, thereby decreasing the production of inflammatory factors and subsequently improving the condition of bones and joints [37]. Notably, our findings indicate that glinides were associated with a reduced risk of heart disease, which is consistent with the results of previous large-scale cohort studies [38]. However, similar outcomes have not been observed in clinical trials investigating glinides agents. We hypothesize that this discrepancy may be attributed to the inhibitory effects of glinides agents on cardiovascular KATP channels, as different insulin secretagogues exhibit varying degrees of selectivity for cardiovascular KATP channels [39].

Furthermore, our findings suggest that T2D patients with hypertension and insulin exposure have a significantly lower risk of developing insomnia disorders compared to those treated with DPP4is and SUs. Several Mendelian randomization studies have identified an association between insomnia disorders and insulin resistance [40, 41]. However, the impact of insulin injection on sleep remains to be elucidated.

With respect to urinary tract infections, acarbose users exhibited the highest risk, which was not unexpected. However, it is noteworthy that GLP-1 RAs users had a significantly lower risk of urinary tract infections, a finding that is consistent with the evidence from previous cohort studies [42]. We also found that insulin users had a higher risk of hepatic failure. We hypothesize that long-term insulin injection may induce or exacerbate insulin resistance, potentially contributing to chronic injury and fibrosis [43].

Overall, our findings provide important insights to inform individualized treatment decisions in patients with T2D and hypertension. In terms of cardiovascular effectiveness, DPP4is, GLP-1 RAs, and glinides consistently outperformed insulin and acarbose, with SUs also showing superiority over insulin but being less effective than DPP4is. These results support prioritizing DPP4is, GLP-1 RAs, and glinides as second-line agents in this high-risk population. However, differences in safety profiles across drug classes provide important considerations for individualized treatment selection. Among these agents, DPP4is notably demonstrated a favorable impact on chronic kidney disease, in addition to their benefits in 3-point MACE and 4-point MACE, reinforcing their value in this high-risk population. Nevertheless, they were also associated with higher risks of coronary atherosclerotic disease and insomnia compared to insulin and other agents, while showing a lower risk of hepatic failure. Compared to DPP4is, glinides were associated with lower risks of coronary atherosclerotic disease, hypertensive heart disease, and urinary tract infections, suggesting potential benefits in patients with cardiovascular or infection susceptibility. Despite these benefits, glinides were also linked to increased risks of chronic kidney disease and inflammatory polyarthritis, indicating they may not be optimal for patients with renal impairment or autoimmune inflammatory conditions. GLP-1 RAs demonstrated a favorable safety profile, particularly with a significantly reduced risk of urinary tract infections compared to acarbose, DPP4is, and SUs. No significant increase in other adverse events was observed, suggesting GLP-1 RAs may be a well-tolerated option for patients with susceptibility to genitourinary infections. Collectively, these findings emphasize the importance of balancing cardiovascular efficacy and safety when selecting second-line therapies, and highlight the value of tailoring treatment choices based on individual comorbidity profiles, including cardiovascular, renal, immune, and neuropsychiatric risks.

This study offers several notable strengths. First, it provides a comprehensive, head-to-head comparison of commonly used novel hypoglycemic agents and traditional therapies within a unified analytical framework, thereby offering robust evidence to support individualized treatment strategies for patients with T2D and hypertension. Second, the study population predominantly comprises Asian individuals, addressing a critical gap in the existing literature related to racial and ethnic representation and enhancing the generalizability of our findings to this understudied population. Third, by leveraging large-scale, real-world electronic health record data, this study complements evidence derived from RCTs. While RCTs, conducted under controlled conditions with selected populations, remain the gold standard for establishing efficacy and causal inference, our analysis provides crucial estimates of real-world effectiveness, reflecting outcomes in routine clinical practice and helping to fill evidence gaps relevant to broader patient populations and heterogeneous healthcare settings. Finally, advanced statistical techniques, including propensity score adjustment, were employed to control for potential confounding factors, thereby strengthening the internal validity and interpretability of the results.

However, there are still some limitations. First, the study lacked data on all-cause mortality and certain clinically important adverse events, such as severe hypoglycemia and fractures, due to institutional data-sharing restrictions, rarity of events, and likely underreporting. This hampers comparability with trial-based survival data and may weaken the robustness of outcome-specific analyses, highlighting the need for further investigation. Second, during the pooled analysis, no relevant outcomes were observed in the SGLT2is group, although their cardiovascular benefits have been confirmed in RCTs. This discrepancy between established efficacy and observed real-world effectiveness warrants further exploration in future investigations. Third, given the relatively limited number of events in the GLP-1 RAs group, particularly in the FAHZU center, the corresponding findings should be interpreted with caution. These results are considered exploratory in nature but may still provide valuable preliminary insights to guide future research. Fourth, although advanced statistical methods were applied to adjust for available covariates, the absence of key clinical factors such as diabetes duration and concomitant medications may have introduced residual confounding. Prospective studies are needed to address these gaps and validate our findings. Therefore, caution is warranted when interpreting the results. In addition, we acknowledge the potential for indication bias, especially in comparisons involving insulin. Since insulin is often prescribed to patients with more advanced or poorly controlled diabetes, their higher cardiovascular risk may reflect underlying disease severity rather than a direct effect of insulin. Despite PSM and statistical adjustments, residual indication bias may persist and should be considered when interpreting these findings. Lastly, these analyses are subject to limitations arising from site-specific treatment patterns and the inherent challenges in accurately ascertaining exposure and outcomes across heterogeneous data sources, which may introduce bias into the observed associations between drug classes and clinical outcomes. Collectively, these limitations highlight the need for large-scale comparative effectiveness studies using harmonized real-world data alongside ongoing RCTs to provide a more complete understanding of treatment outcomes.

## Conclusion

Our large, rigorous, multinational network study of patients with T2D and hypertension demonstrated that DPP4is, GLP-1 RAs, and glinides significantly reduced cardiovascular risk. All three agents were more effective than insulin and acarbose. While SUs outperformed insulin, they were less effective than DPP4is. No statistically significant differences were observed among the other drug groups. These findings highlight the importance of prioritizing DPP4is, GLP-1 RAs, and glinides as second-line agents for T2D patients with hypertension.

## Supplementary Information

Below is the link to the electronic supplementary material.

Supplementary Material 1

Supplementary Material 2

## Data Availability

The data that support the findings of this study are available from JSPH but restrictions apply to the availability of these data, which were used under license for the current study, and therefore are not publicly available. Data are however available from the co-authors upon reasonable request and with permission of JSPH.

## Abbreviations

T2D: Type 2 diabetes
SGLT2is: Sodium-glucose transporter 2 inhibitors
GLP-1 RAs: Glucagon-like peptide-1 receptor agonists
DPP4is: Dipeptidyl peptidase-4 inhibitors
SUs: Sulfonylureas
MACE: Major adverse cardiovascular events
MI: Myocardial infarction
HF: Heart failure
SCD: Sudden cardiac death
RCTs: Randomized controlled trials
ADA: American diabetes association
ESC: European society of cardiology
EASD: European association for the study of diabetes
OHDSI: Observational health data science and informatics
EHR: Electronic health record
OMOP: Observational medical outcome partnership
CDM: Common data model
JSPH: Jiangsu Provincial people's hospital
FAHZU: The first affiliated hospital, Zhejiang university school of medicine
PSM: Propensity score matching
IPTW: Inverse probability of treatment weighting
SD: Standard deviation
SMD: Standardized mean difference
HR: Hazard ratio
CI: Confidence interval
FDR: False discovery rate
