# Supplementary material for: Comparative cardiovascular outcomes and safety of hypoglycemic drug classes in patients with type 2 diabetes and hypertension: a multicenter cohort analysis
Source: Cardiovasc Diabetol. 2025 Aug 20;24:343. doi: 10.1186/s12933-025-02892-5 (PMC12369064; doi:10.1186/s12933-025-02892-5)
Supplement: Supplementary file 1 — Supplementary Material 1 [file 12933_2025_2892_MOESM1_ESM.pdf]

Supplementary figures

SFigure 1a-b: Propensity score distributions for hypoglycemic drug comparisons before adjustment using PSM and IPTW methods in the JSPH and FAHZU Database....1
SFigure 2a-d: Demographic characteristic balance across hypoglycemic drug classes before and after adjustment using PSM and IPTW methods in the JSPH and FAHZU Database....3
SFigure 3a-u: Detailed demographic characteristic balance for each hypoglycemic drug comparison before and after adjustment using PSM and IPTW methods in the JSPH and FAHZU databases....7
SFigure 4a-u: Survival curves for 3-point and 4-point MACE across hypoglycemic drug classes using PSM and IPTW methods in the JSPH and FAHZU Databases.....17
SFigure 5: Comparative effectiveness of hypoglycemic drug classes on seven cardiovascular events using PSM method in JSPH, FAHZU, and Pooled analysis....28
SFigure 6: Comparative safety profiles of hypoglycemic drug classes on ten events using PSM method in JSPH, FAHZU, and Pooled analysis....29
SFigure 7a-c: Sensitivity analysis of hypoglycemic drug classes on seven cardiovascular events using PSM and IPTW methods in the JSPH, FAHZU, and Pooled analysis....30
SFigure 8a-c: Sensitivity analysis of hypoglycemic drug classes on ten events using PSM and IPTW methods in the JSPH, FAHZU, and Pooled analysis 33

List of Abbreviations Used in the Supplementary Figures

Abbreviation	Definition
PSM	propensity score matching
IPTW	inverse probability of treatment weighting
JSPH	Jiangsu Provincial People's Hospital
FAHZU	The First Affiliated Hospital, Zhejiang University School of Medicine
HR	hazard ratio
CI	confidence interval
SMD	standardized mean differences
MACE	major adverse cardiovascular events
MetInsulin	metformin combined with insulin
MetSUss	metformin combined with sulfonylureas
MetAcarbose	metformin combined with acarbose
MetSGLT2iss	metformin combined with sodium-glucose transporter 2 inhibitors
MetGLP-1 RAs	metformin combined with glucagon-like peptide-1 receptor agonists
MetGlinides	metformin combined with glinides
MetDPP4iss	metformin combined with dipeptidyl peptidase-4 inhibitors

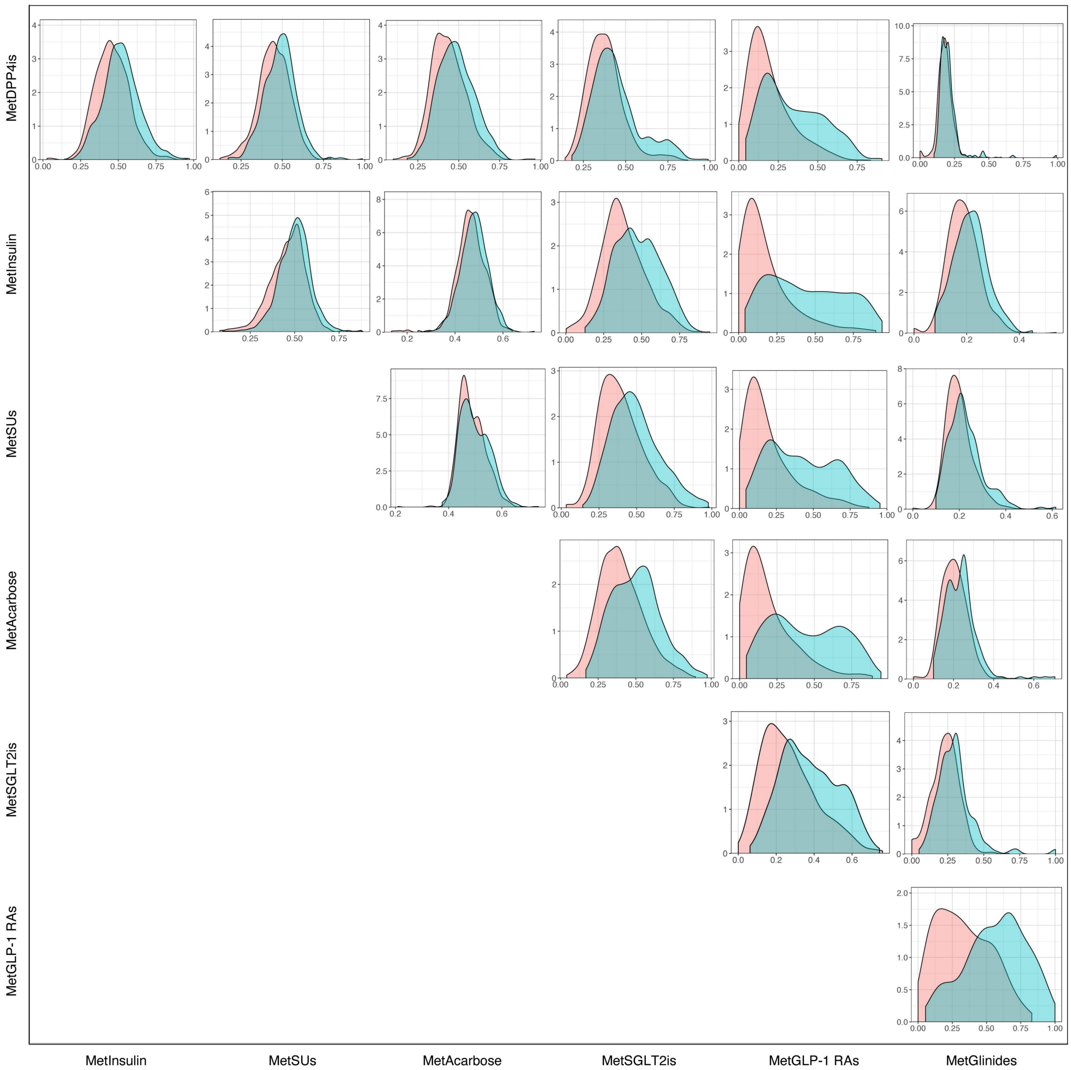

SFigure 1a: Propensity score distributions for hypoglycemic drug comparisons before adjustment using PSM and IPTW methods in the JSPH Database. For each drug comparison, the target drug class is represented in the row, while the comparator drug class is represented in the column. Blue curves represent the propensity score distribution for the target drug class, and red curves represent the propensity score distribution for the comparator drug class. The x-axis represents the propensity scores, while the y-axis represents the density of observations.

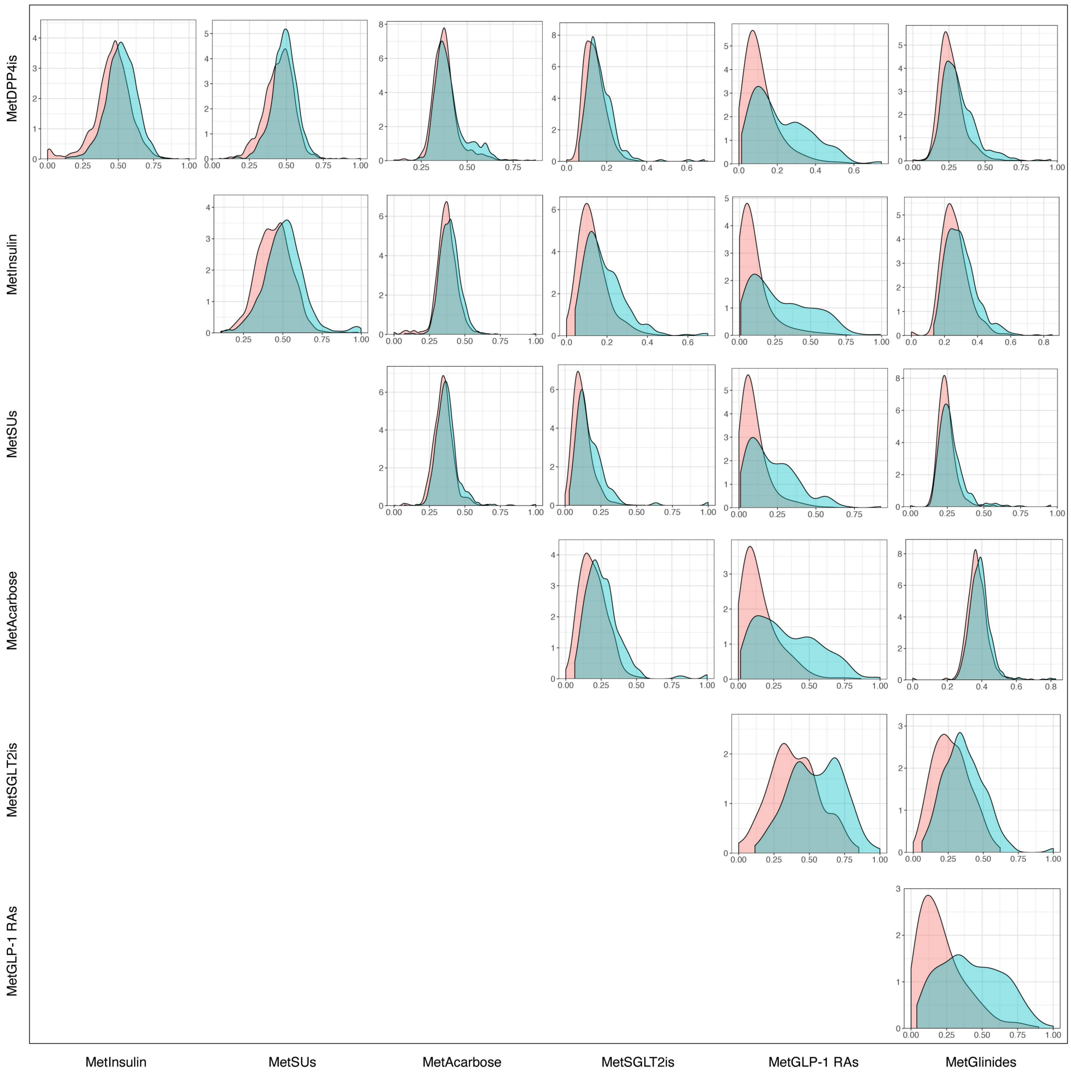

SFigure 1b: Propensity score distributions for hypoglycemic drug comparisons before adjustment using PSM and IPTW methods in the FAHZU Database. For each drug comparison, the target drug class is represented in the row, while the comparator drug class is represented in the column. Blue curves represent the propensity score distribution for the target drug class, and red curves represent the propensity score distribution for the comparator drug class. The x-axis represents the propensity scores, while the y-axis represents the density of observations.

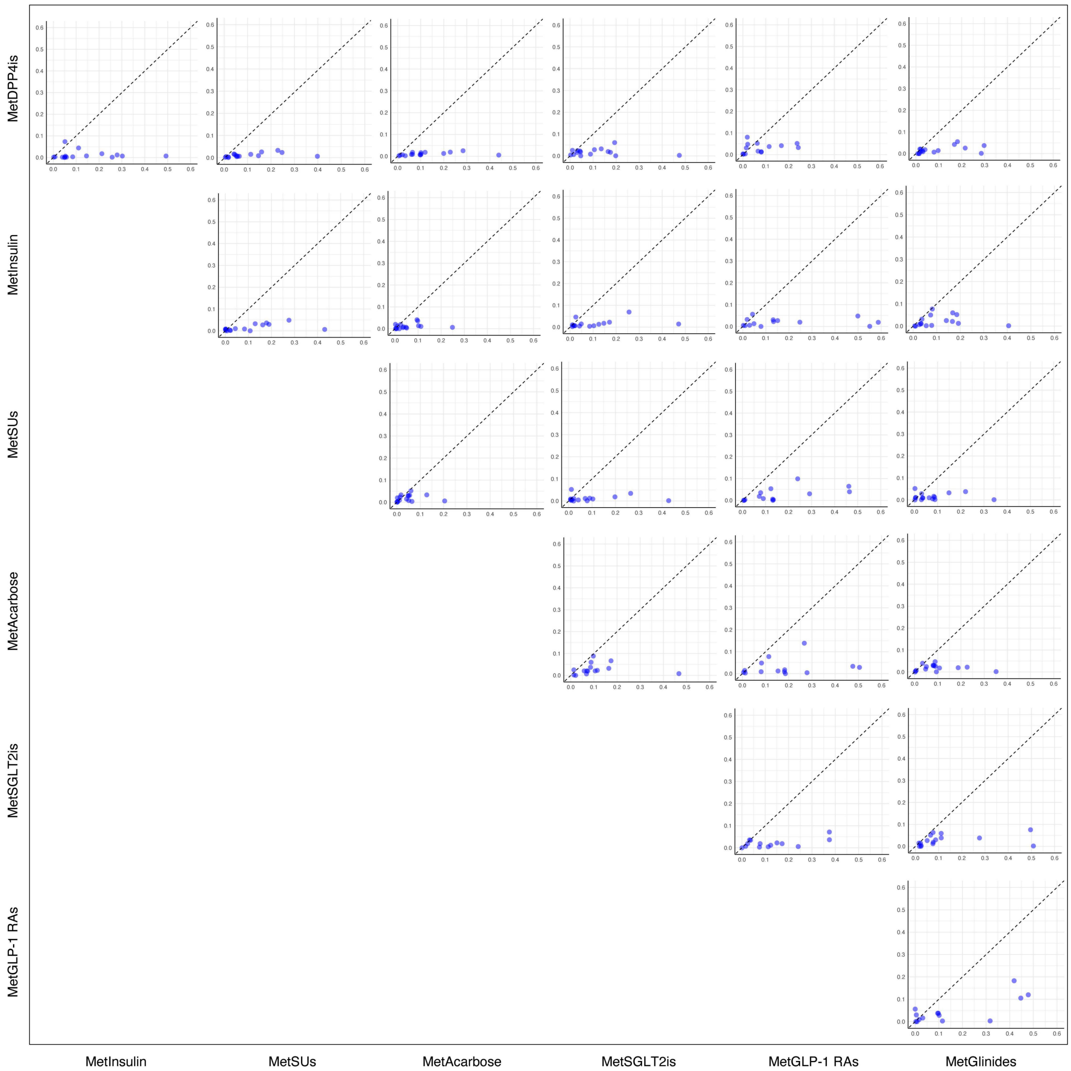

SFigure 2a: Demographic characteristic balance across hypoglycemic drug classes before and after adjustment using PSM method in the JSPH Database. For each drug comparison, the target drug class is represented in the row, while the comparator drug class is represented in the column. Each blue dot represents the SMD for a single covariate, plotted before PSM on the x-axis and after PSM on the y-axis. An SMD value of < 0.1 is considered indicative of acceptable balance.

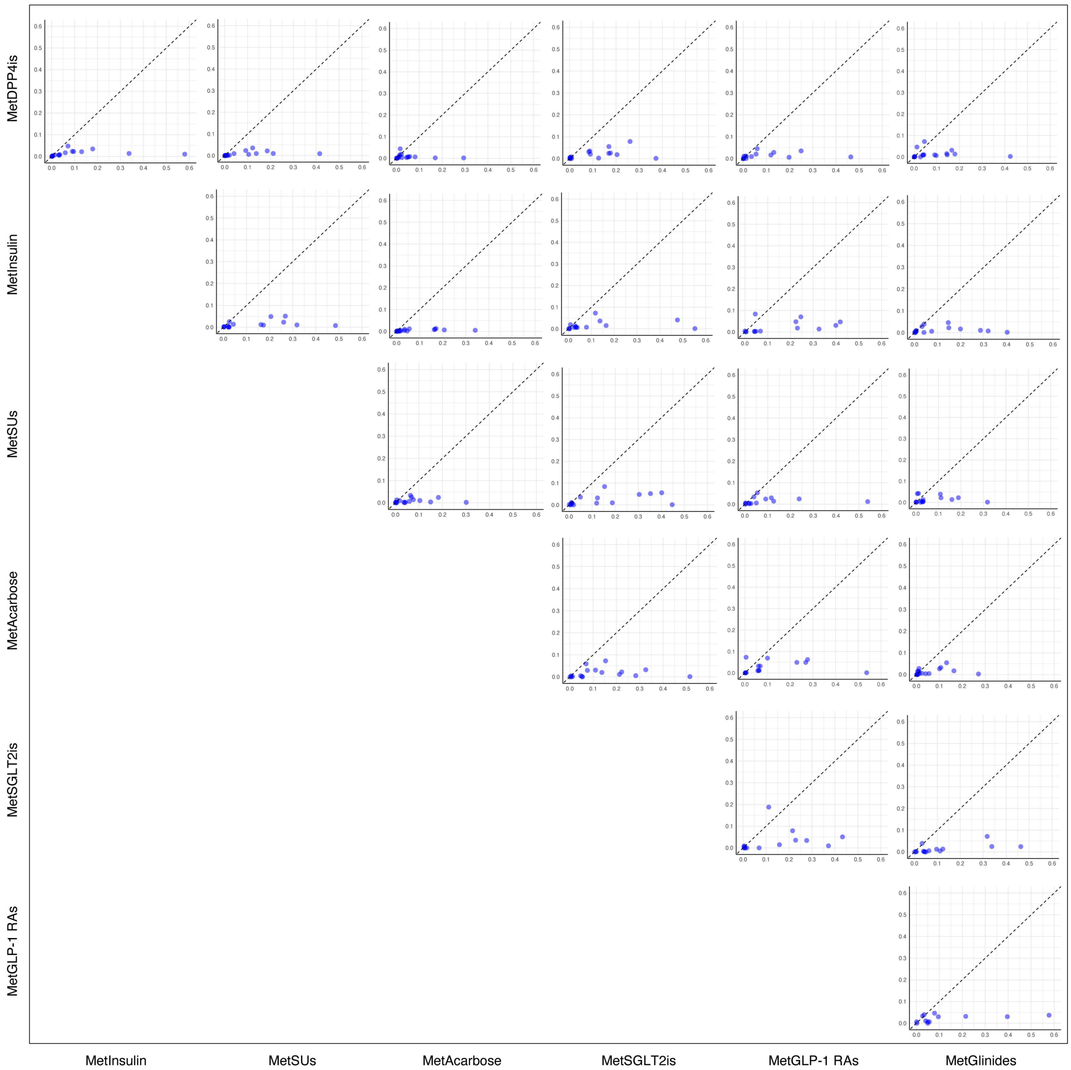

SFigure 2b: Demographic characteristic balance across hypoglycemic drug classes before and after adjustment using PSM method in the FAHZU Database. For each drug comparison, the target drug class is represented in the row, while the comparator drug class is represented in the column. Each blue dot represents the SMD for a single covariate, plotted before PSM on the x-axis and after PSM on the y-axis. An SMD value of < 0.1 is considered indicative of acceptable balance.

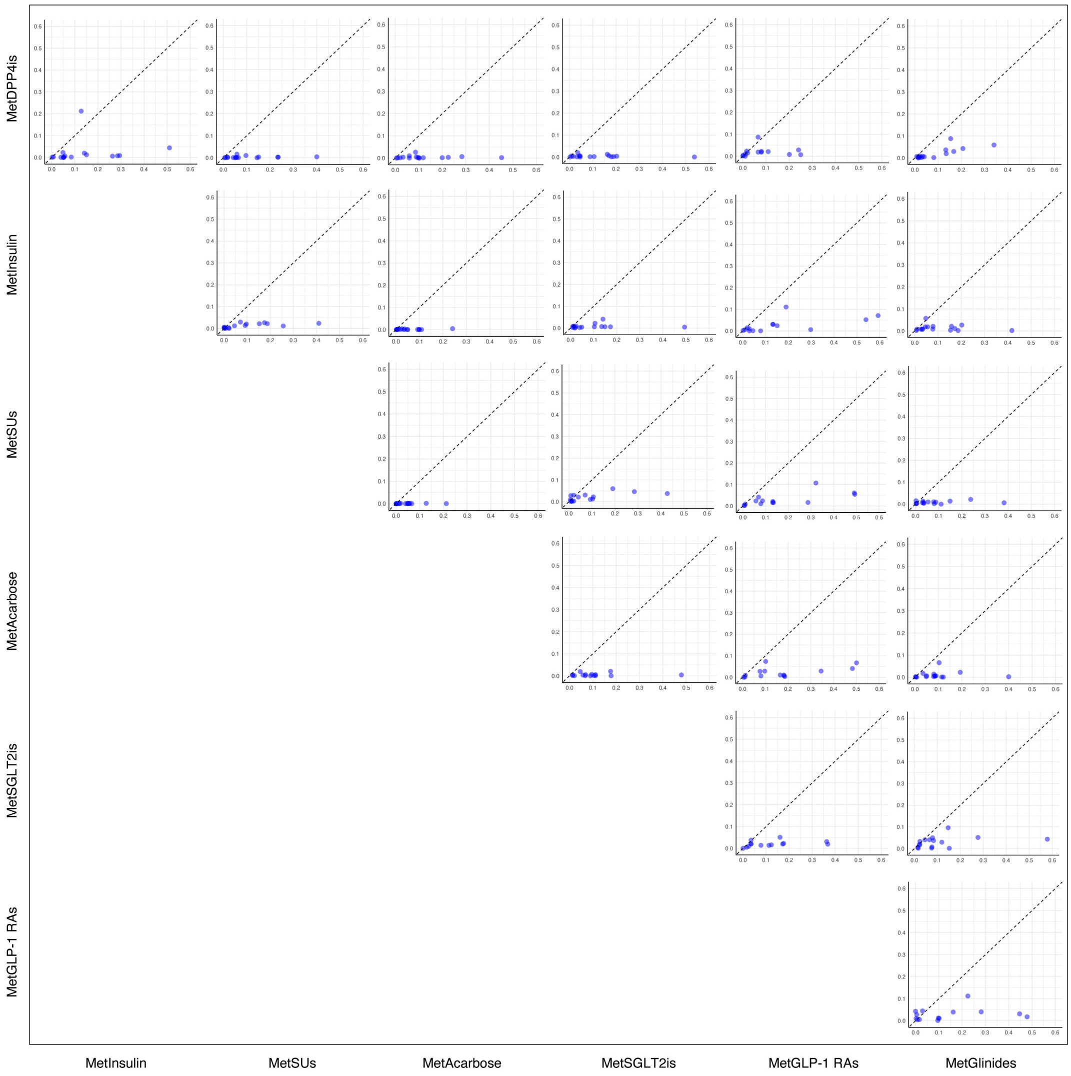

SFigure 2c: Demographic characteristic balance across hypoglycemic drug classes before and after adjustment using IPTW method in the JSPH Database. For each drug comparison, the target drug class is represented in the row, while the comparator drug class is represented in the column. Each blue dot represents the SMD for a single covariate, plotted before PSM on the x-axis and after PSM on the y-axis. An SMD value of < 0.1 is considered indicative of acceptable balance.

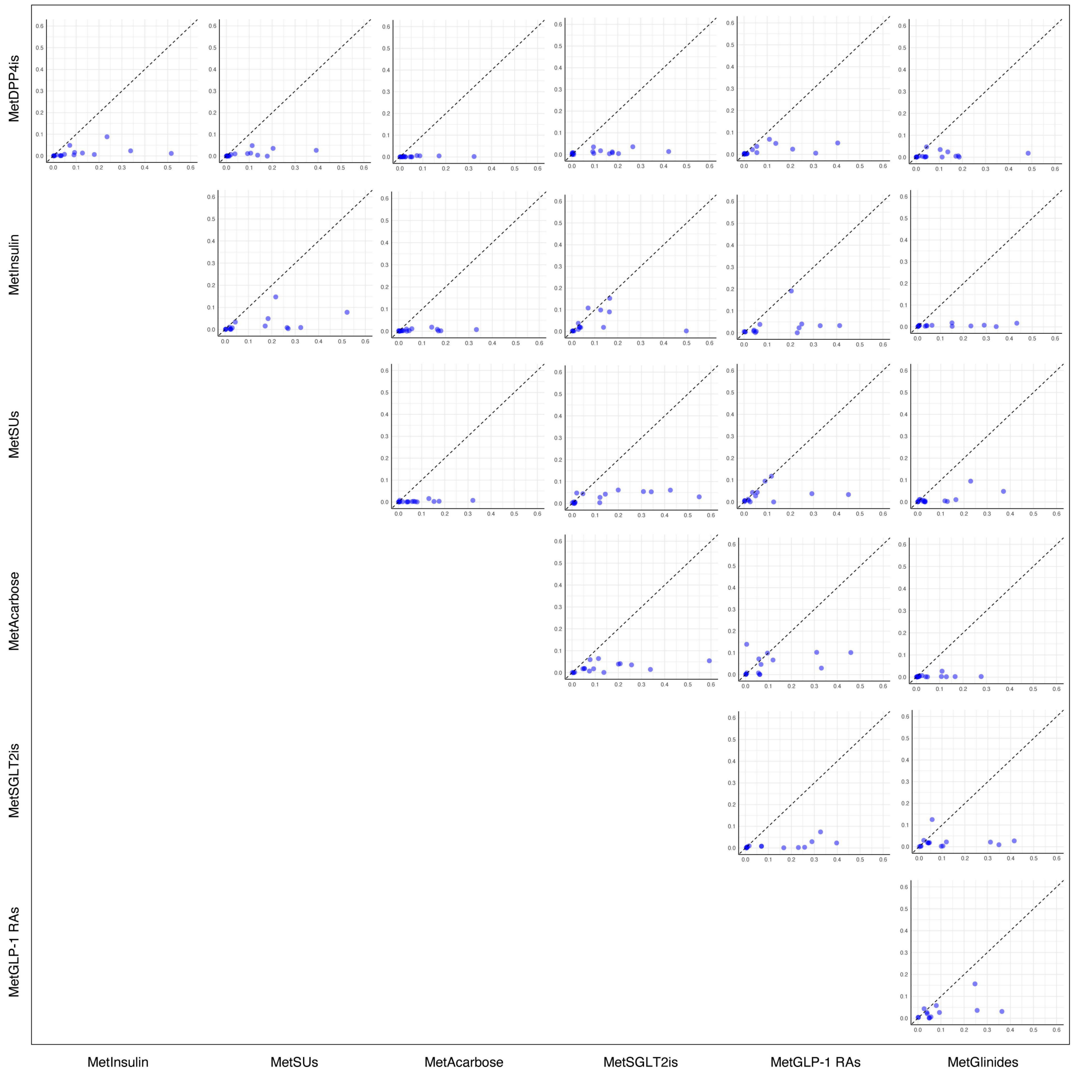

SFigure 2d: Demographic characteristic balance across hypoglycemic drug classes before and after adjustment using IPTW method in the FAHZU Database. For each drug comparison, the target drug class is represented in the row, while the comparator drug class is represented in the column. Each blue dot represents the SMD for a single covariate, plotted before PSM on the x-axis and after PSM on the y-axis. An SMD value of < 0.1 is considered indicative of acceptable balance.

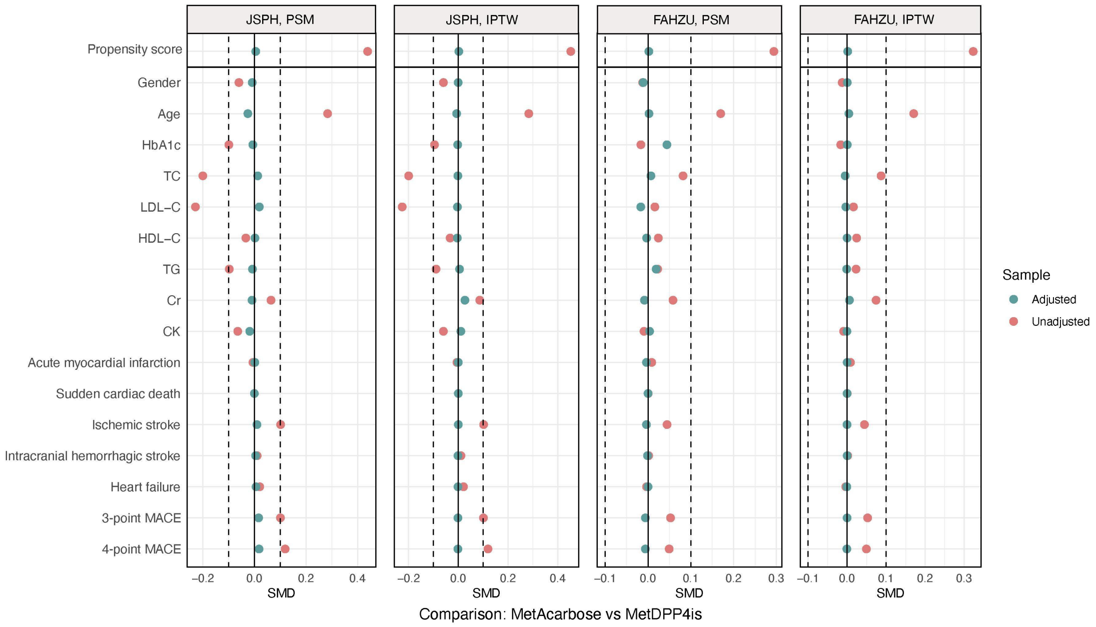

SFigure 3a: Demographic characteristic balance for MetAcarbose and MetDPP4is before and after adjustment using PSM and IPTW methods in the JSPH and FAHZU databases. Red lines represent SMDs before adjustment (unadjusted), and blue lines represent SMDs after adjustment (adjusted). An SMD value within ±0.1 is considered indicative of acceptable balance.

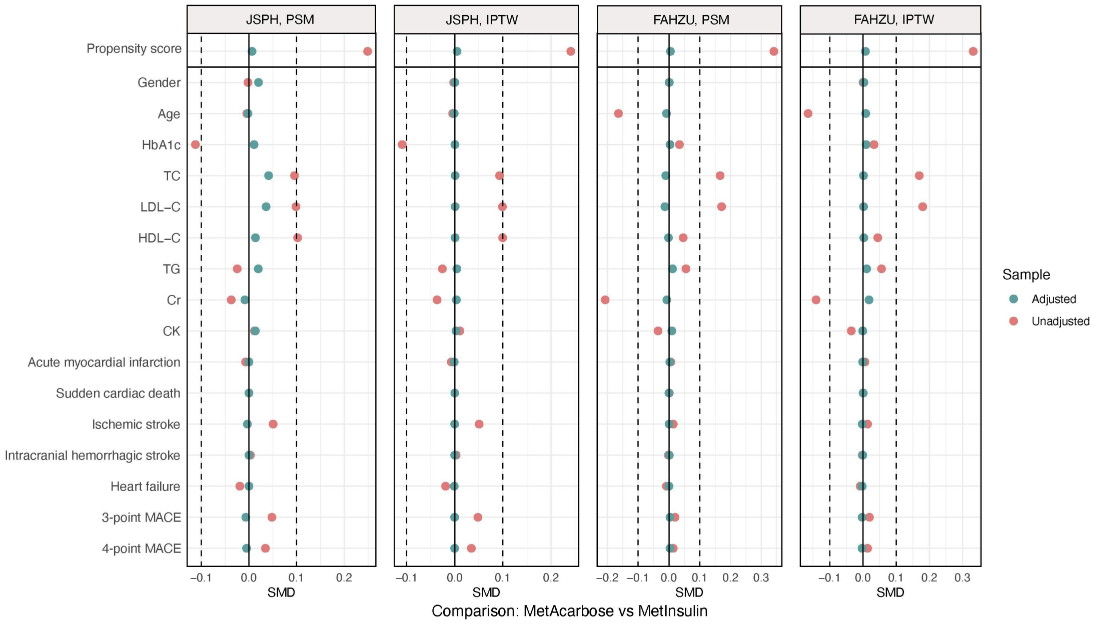

SFigure 3b: Demographic characteristic balance for MetAcarbose and MetInsulin before and after adjustment using PSM and IPTW methods in the JSPH and FAHZU databases. Red lines represent SMDs before adjustment (unadjusted), and blue lines represent SMDs after adjustment (adjusted). An SMD value within ±0.1 is considered indicative of acceptable balance.

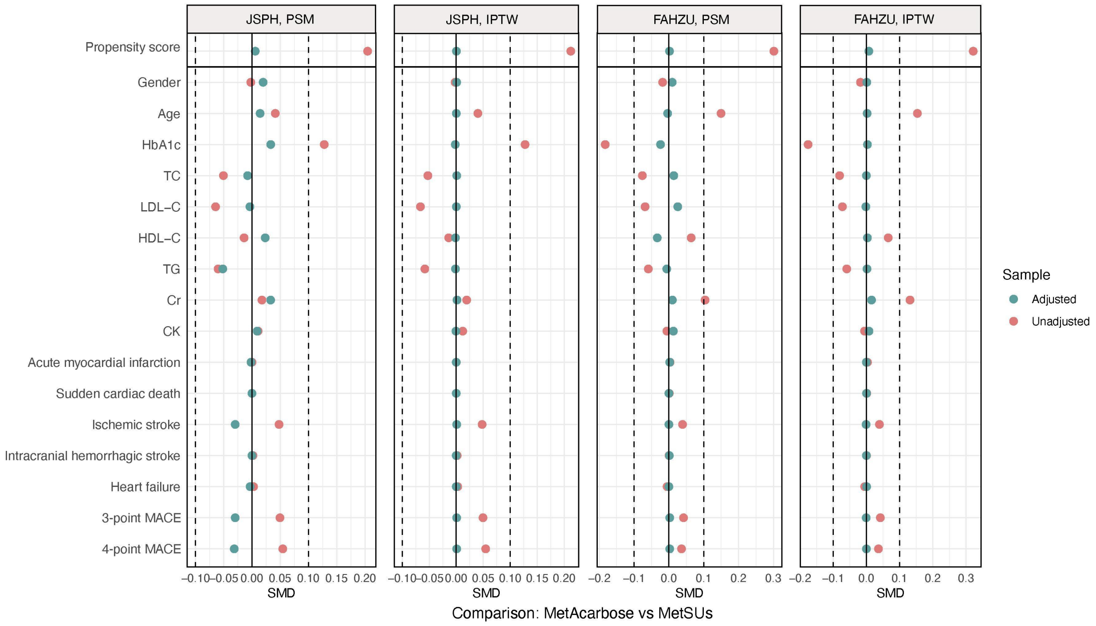

SFigure 3c: Demographic characteristic balance for MetAcarbose and MetSUs before and after adjustment using PSM and IPTW methods in the JSPH and FAHZU databases. Red lines represent SMDs before adjustment (unadjusted), and blue lines represent SMDs after adjustment (adjusted). An SMD value within ±0.1 is considered indicative of acceptable balance.

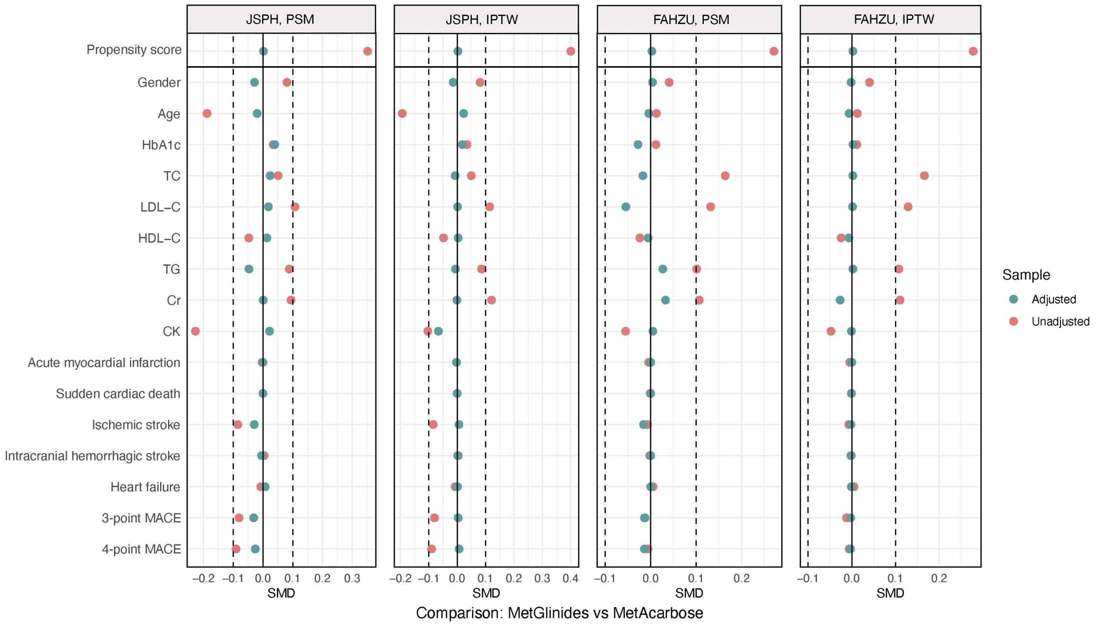

SFigure 3d: Demographic characteristic balance for MetGlinides and MetAcarbose before and after adjustment using PSM and IPTW methods in the JSPH and FAHZU databases. Red lines represent SMDs before adjustment (unadjusted), and blue lines represent SMDs after adjustment (adjusted). An SMD value within ±0.1 is considered indicative of acceptable balance.

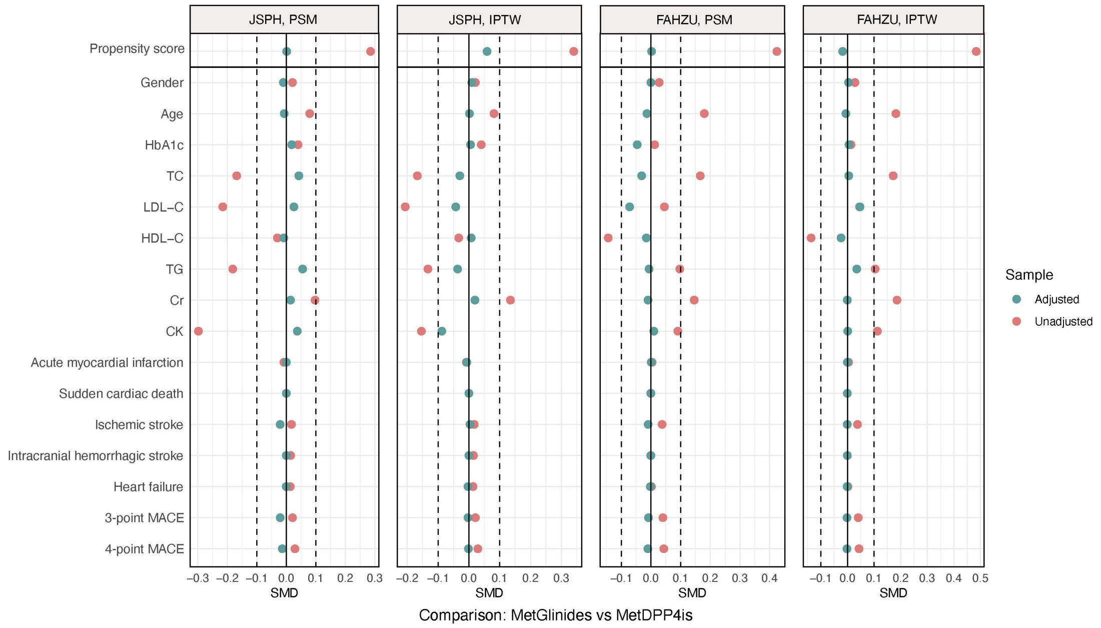

SFigure 3e: Demographic characteristic balance for MetGlinides and MetDPP4is before and after adjustment using PSM and IPTW methods in the JSPH and FAHZU databases. Red lines represent SMDs before adjustment (unadjusted), and blue lines represent SMDs after adjustment (adjusted). An SMD value within ±0.1 is considered indicative of acceptable balance.

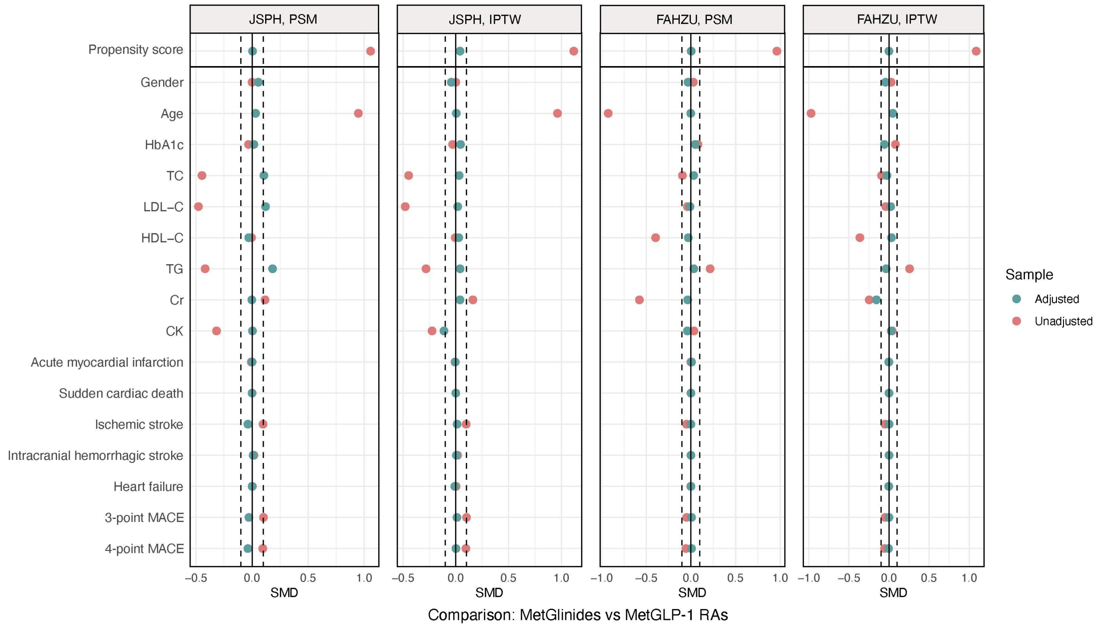

SFigure 3f: Demographic characteristic balance for MetGlinides and MetGLP-1 RAs before and after adjustment using PSM and IPTW methods in the JSPH and FAHZU databases. Red lines represent SMDs before adjustment (unadjusted), and blue lines represent SMDs after adjustment (adjusted). An SMD value within ± 0.1 is considered indicative of acceptable balance.

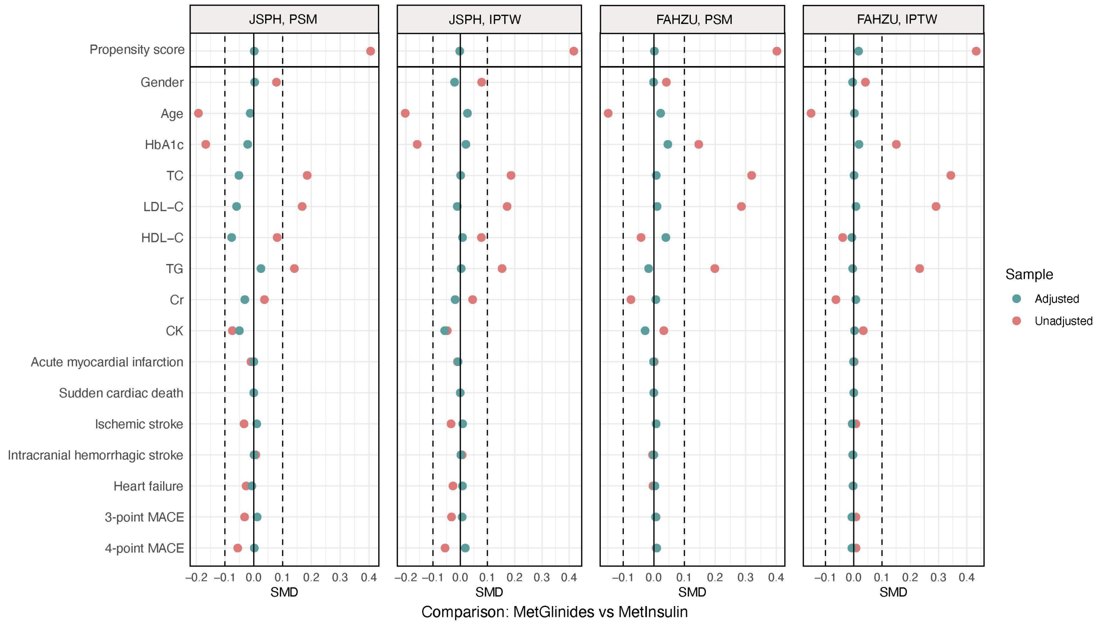

SFigure 3g: Demographic characteristic balance for MetGlinides and MetInsulin before and after adjustment using PSM and IPTW methods in the JSPH and FAHZU databases. Red lines represent SMDs before adjustment (unadjusted), and blue lines represent SMDs after adjustment (adjusted). An SMD value within ±0.1 is considered indicative of acceptable balance.

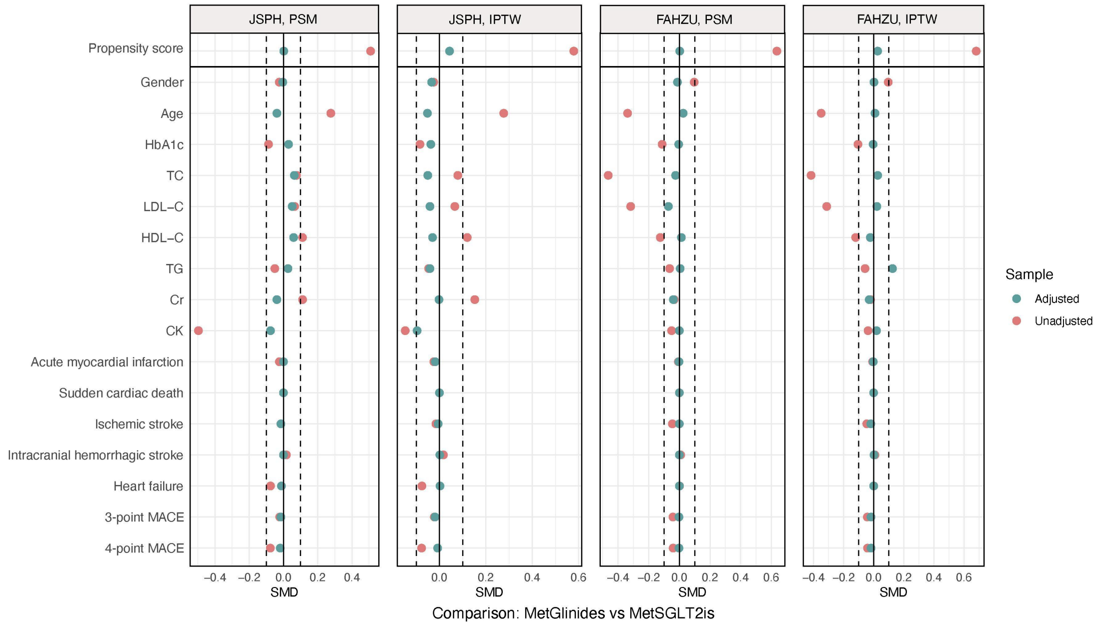

SFigure 3h: Demographic characteristic balance for MetGlinides and MetSGLT2is before and after adjustment using PSM and IPTW methods in the JSPH and FAHZU databases. Red lines represent SMDs before adjustment (unadjusted), and blue lines represent SMDs after adjustment (adjusted). An SMD value within ±0.1 is considered indicative of acceptable balance.

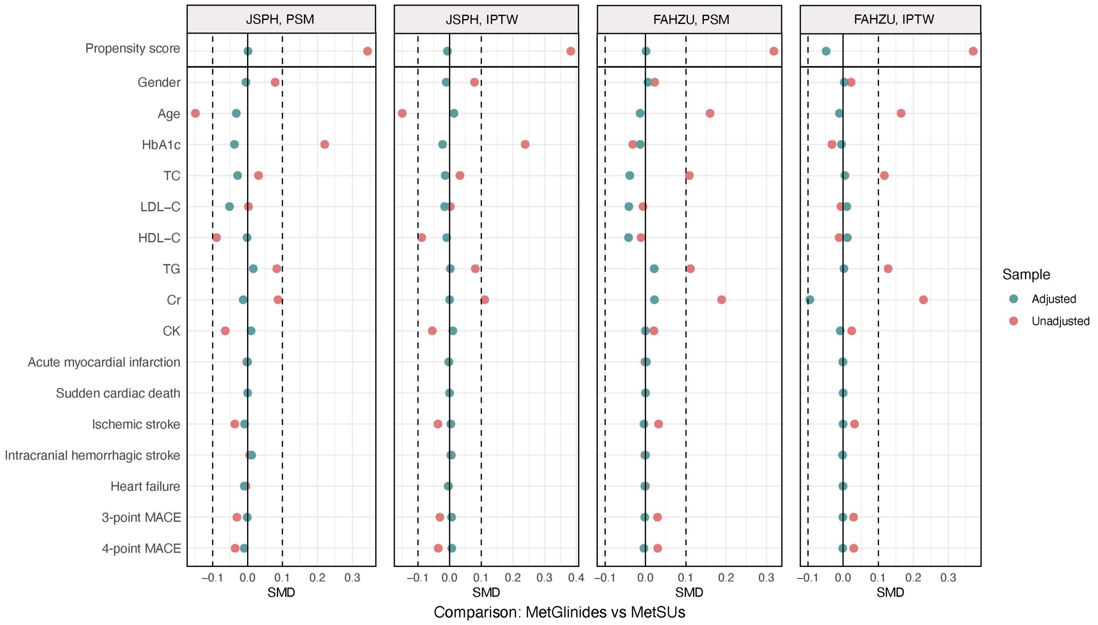

SFigure 3i: Demographic characteristic balance for MetGlinides and MetSUs before and after adjustment using PSM and IPTW methods in the JSPH and FAHZU databases. Red lines represent SMDs before adjustment (unadjusted), and blue lines represent SMDs after adjustment (adjusted). An SMD value within ±0.1 is considered indicative of acceptable balance.

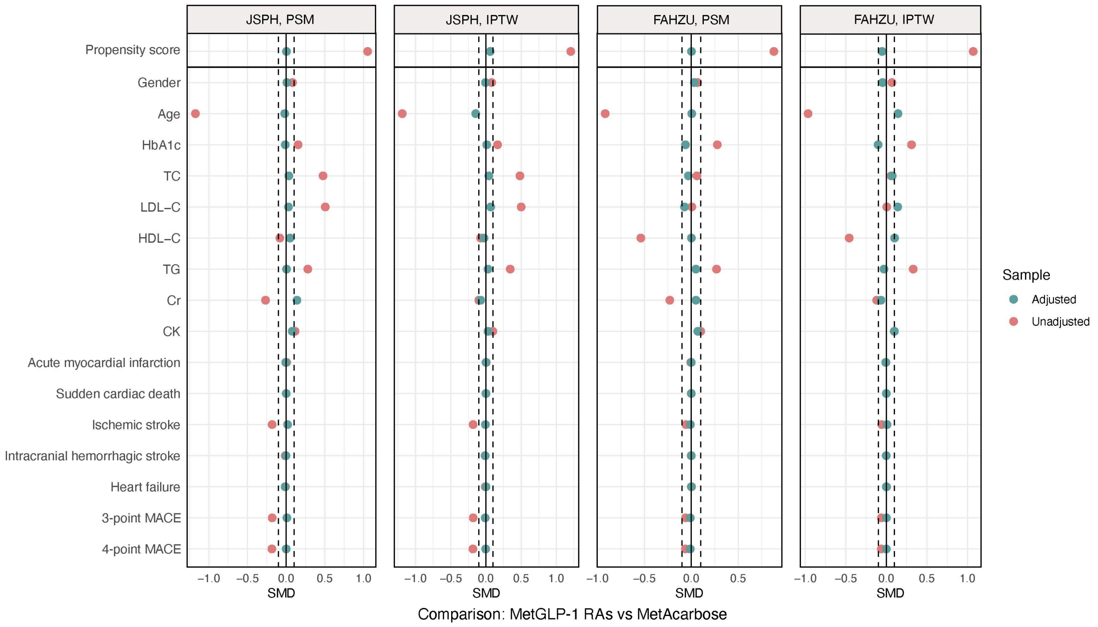

SFigure 3j: Demographic characteristic balance for MetGLP-1 RAs and MetAcarbose before and after adjustment using PSM and IPTW methods in the JSPH and FAHZU databases. Red lines represent SMDs before adjustment (unadjusted), and blue lines represent SMDs after adjustment (adjusted). An SMD value within ±0.1 is considered indicative of acceptable balance.

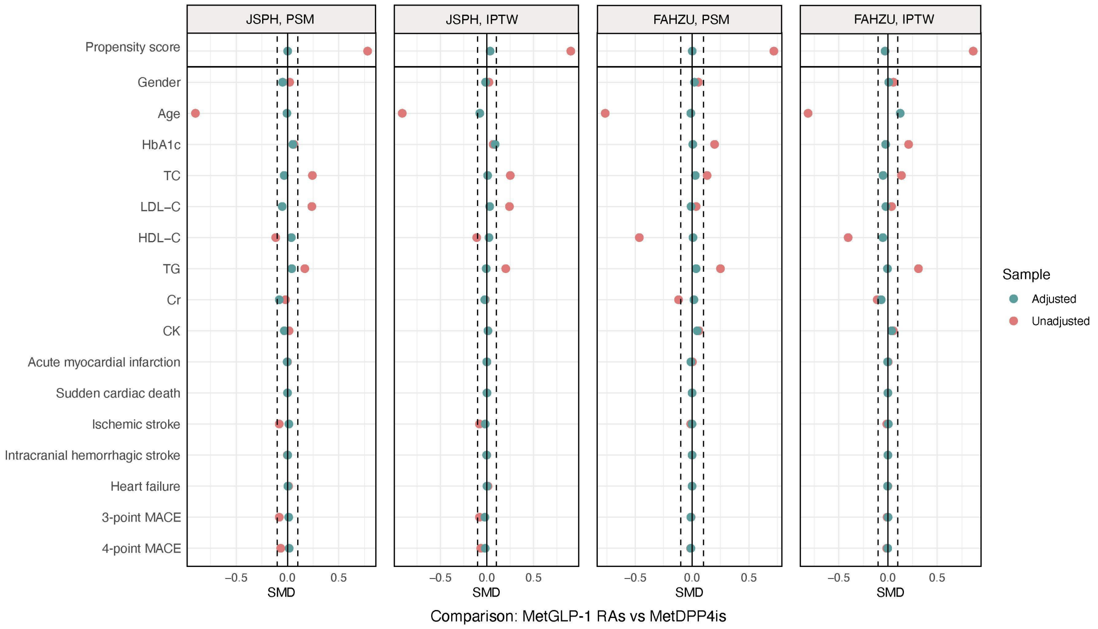

SFigure 3k: Demographic characteristic balance for MetGLP-1 RAs and MetDPP4is before and after adjustment using PSM and IPTW methods in the JSPH and FAHZU databases. Red lines represent SMDs before adjustment (unadjusted), and blue lines represent SMDs after adjustment (adjusted). An SMD value within ±0.1 is considered indicative of acceptable balance.

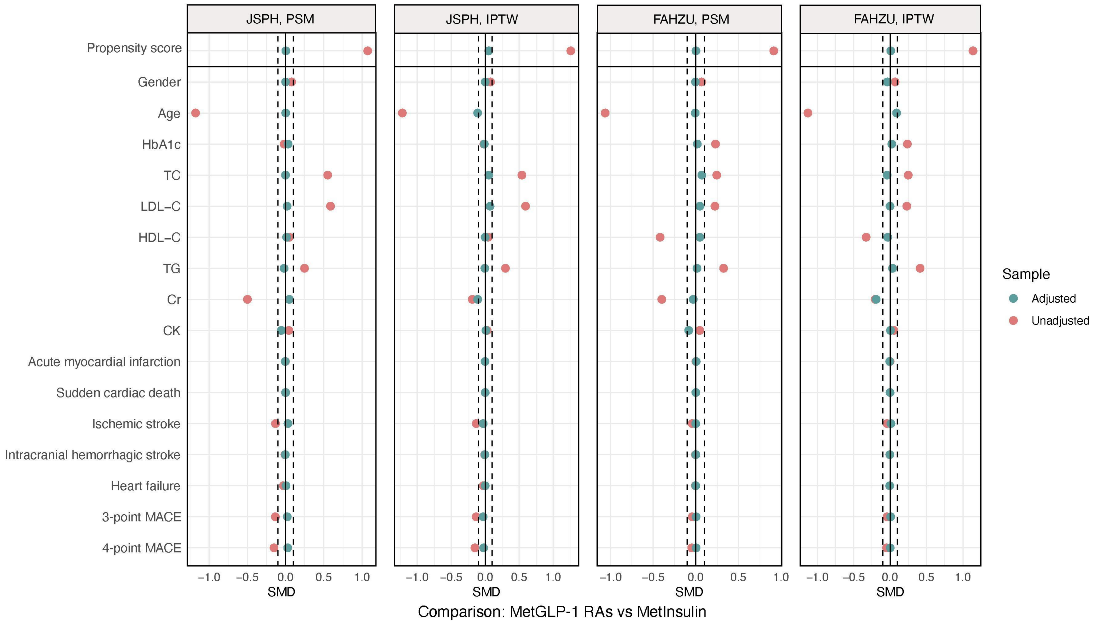

SFigure 3l: Demographic characteristic balance for MetGLP-1 RAs and MetInsulin before and after adjustment using PSM and IPTW methods in the JSPH and FAHZU databases. Red lines represent SMDs before adjustment (unadjusted), and blue lines represent SMDs after adjustment (adjusted). An SMD value within ±0.1 is considered indicative of acceptable balance.

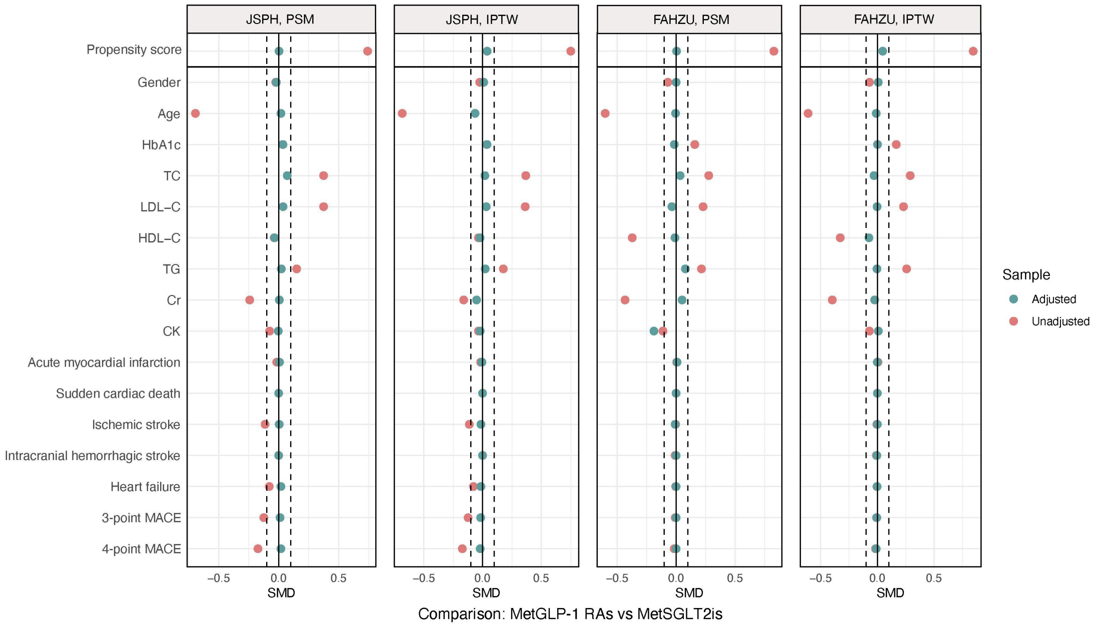

SFigure 3m: Demographic characteristic balance for MetGLP-1 RAs and MetSGLT2is before and after adjustment using PSM and IPTW methods in the JSPH and FAHZU databases. Red lines represent SMDs before adjustment (unadjusted), and blue lines represent SMDs after adjustment (adjusted). An SMD value within ±0.1 is considered indicative of acceptable balance.

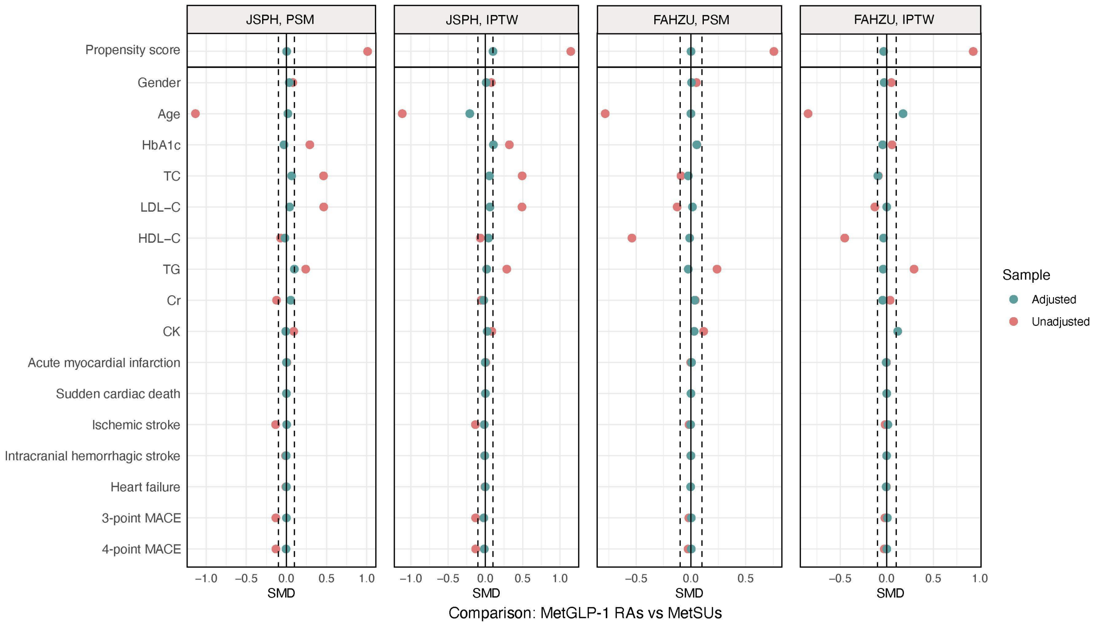

SFigure 3n: Demographic characteristic balance for MetGLP-1 RAs and MetSUs before and after adjustment using PSM and IPTW methods in the JSPH and FAHZU databases. Red lines represent SMDs before adjustment (unadjusted), and blue lines represent SMDs after adjustment (adjusted). An SMD value within ±0.1 is considered indicative of acceptable balance.

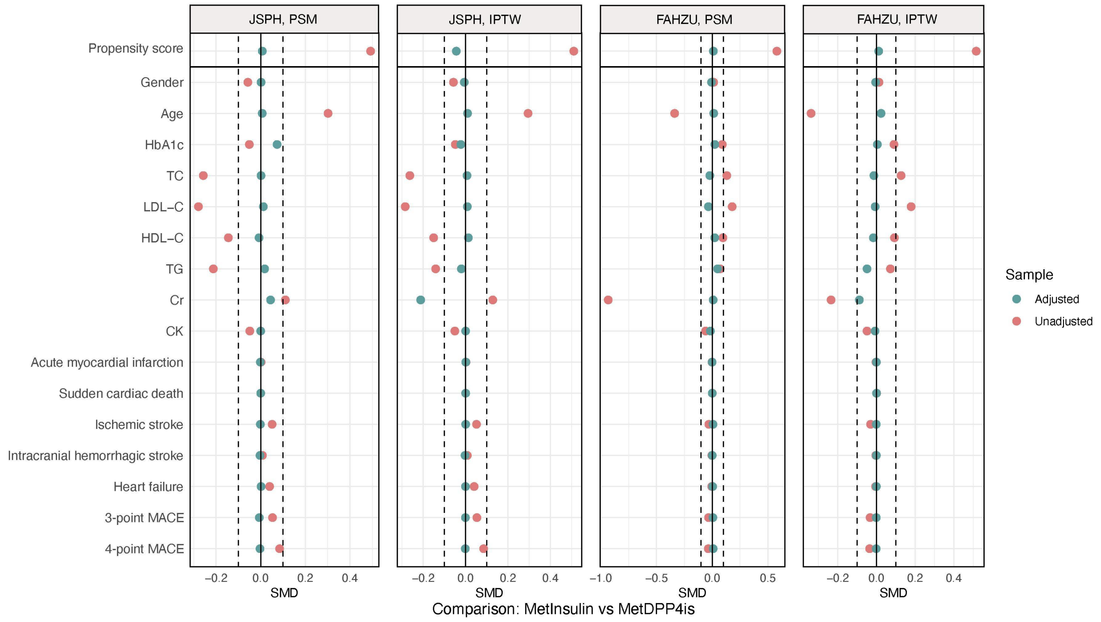

SFigure 3o: Demographic characteristic balance for MetInsulin and MetDPP4is before and after adjustment using PSM and IPTW methods in the JSPH and FAHZU databases. Red lines represent SMDs before adjustment (unadjusted), and blue lines represent SMDs after adjustment (adjusted). An SMD value within ±0.1 is considered indicative of acceptable balance.

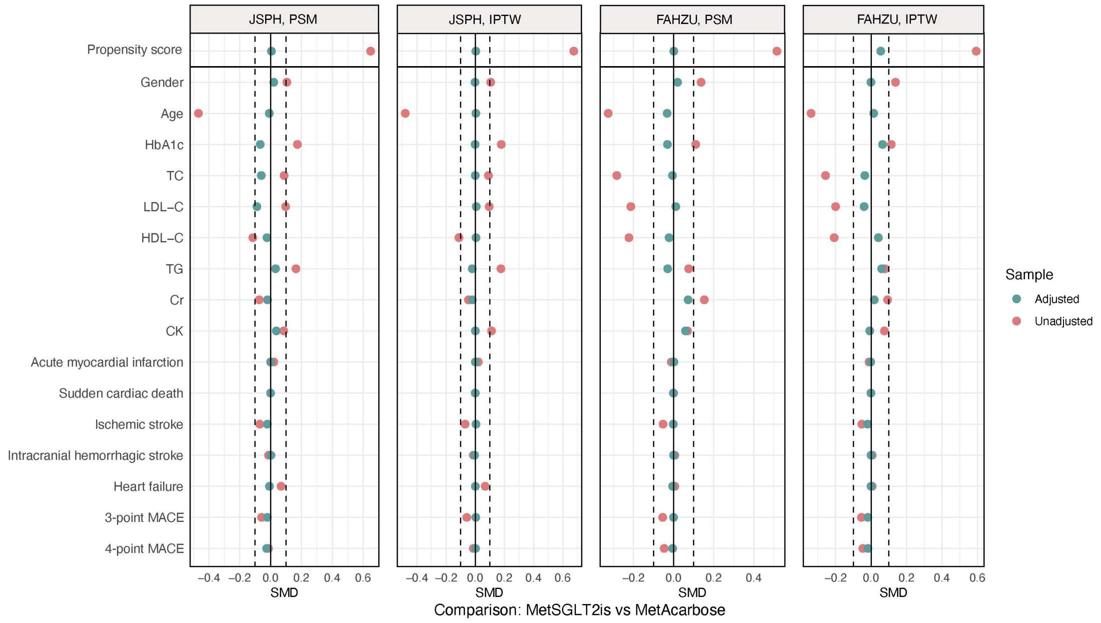

SFigure 3p: Demographic characteristic balance for MetSGLT2is and MetAcarbose before and after adjustment using PSM and IPTW methods in the JSPH and FAHZU databases. Red lines represent SMDs before adjustment (unadjusted), and blue lines represent SMDs after adjustment (adjusted). An SMD value within ±0.1 is considered indicative of acceptable balance.

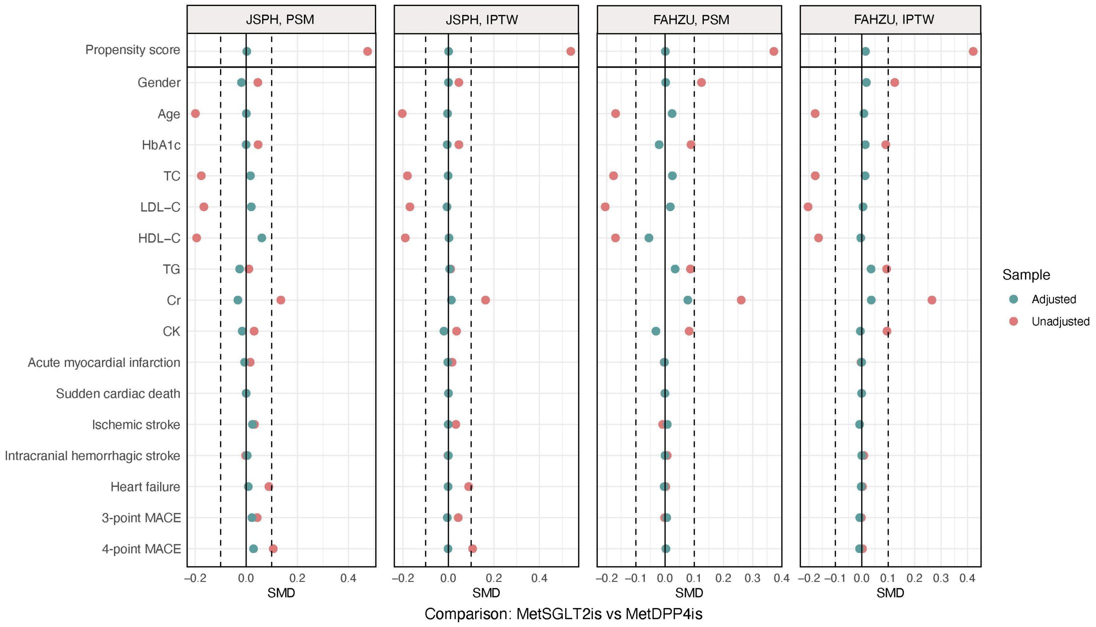

SFigure 3q: Demographic characteristic balance for MetSGLT2is and MetDPP4is before and after adjustment using PSM and IPTW methods in the JSPH and FAHZU databases. Red lines represent SMDs before adjustment (unadjusted), and blue lines represent SMDs after adjustment (adjusted). An SMD value within ±0.1 is considered indicative of acceptable balance.

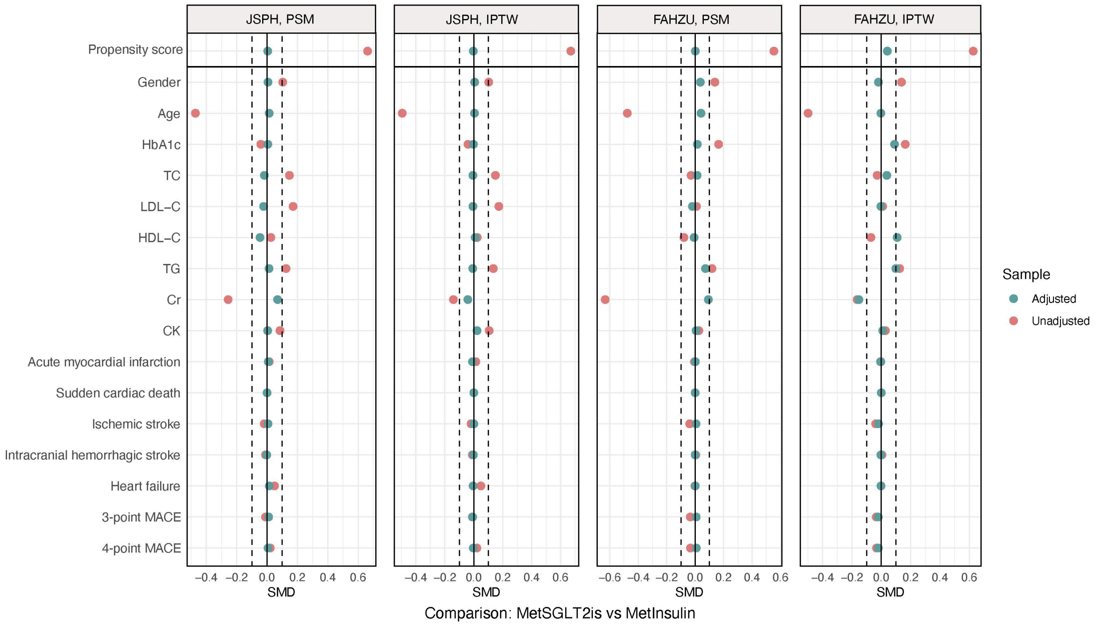

SFigure 3r: Demographic characteristic balance for MetSGLT2is and MetInsulin before and after adjustment using PSM and IPTW methods in the JSPH and FAHZU databases. Red lines represent SMDs before adjustment (unadjusted), and blue lines represent SMDs after adjustment (adjusted). An SMD value within ±0.1 is considered indicative of acceptable balance.

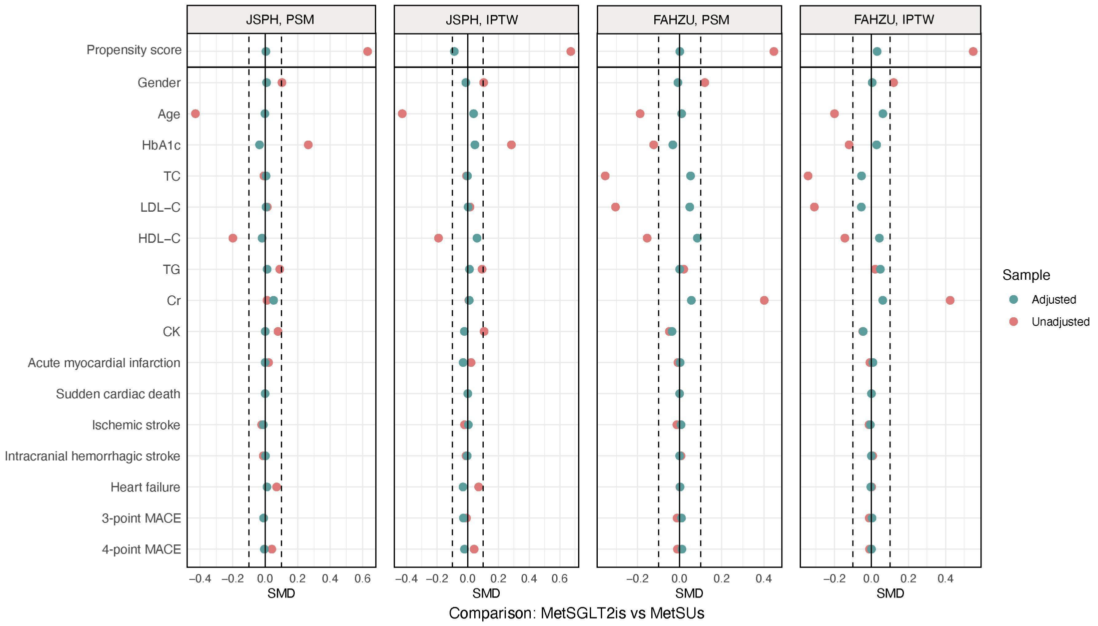

SFigure 3s: Demographic characteristic balance for MetSGLT2is and MetSUs before and after adjustment using PSM and IPTW methods in the JSPH and FAHZU databases. Red lines represent SMDs before adjustment (unadjusted), and blue lines represent SMDs after adjustment (adjusted). An SMD value within ±0.1 is considered indicative of acceptable balance.

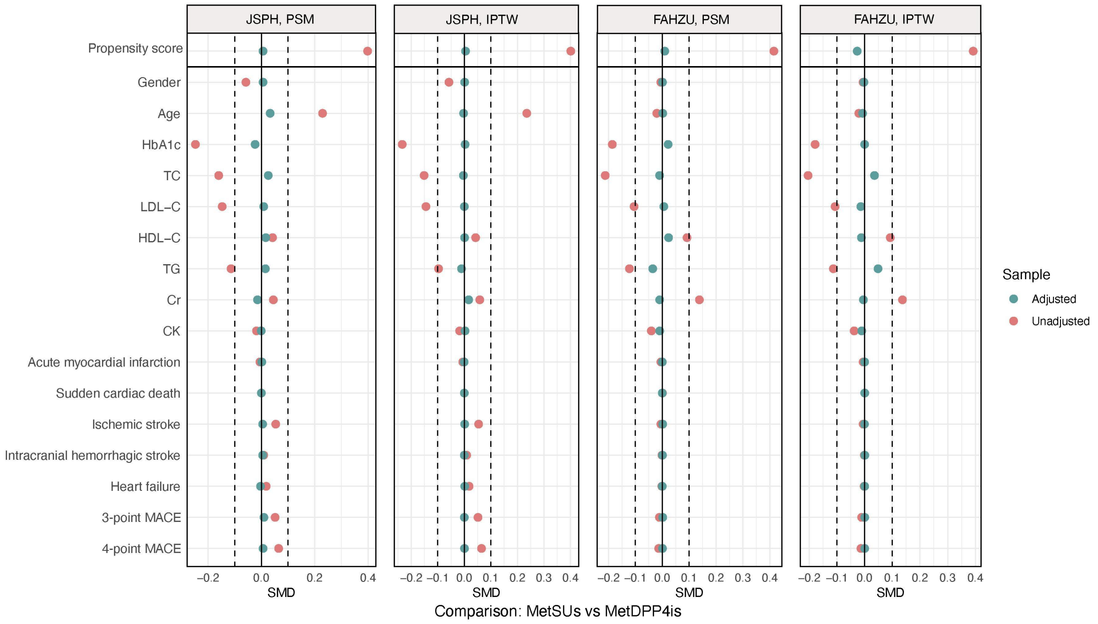

SFigure 3t: Demographic characteristic balance for MetSUs and MetDPP4is before and after adjustment using PSM and IPTW methods in the JSPH and FAHZU databases. Red lines represent SMDs before adjustment (unadjusted), and blue lines represent SMDs after adjustment (adjusted). An SMD value within ±0.1 is considered indicative of acceptable balance.

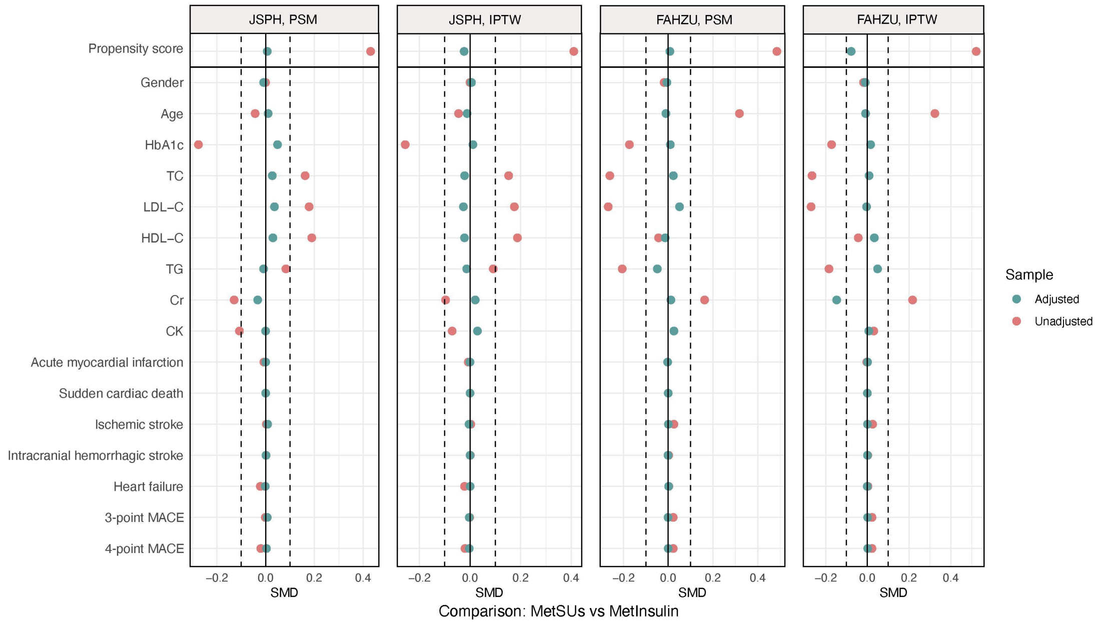

SFigure 3u: Demographic characteristic balance for MetSUs and MetInsulin before and after adjustment using PSM and IPTW methods in the JSPH and FAHZU databases. Red lines represent SMDs before adjustment (unadjusted), and blue lines represent SMDs after adjustment (adjusted). An SMD value within ±0.1 is considered indicative of acceptable balance.

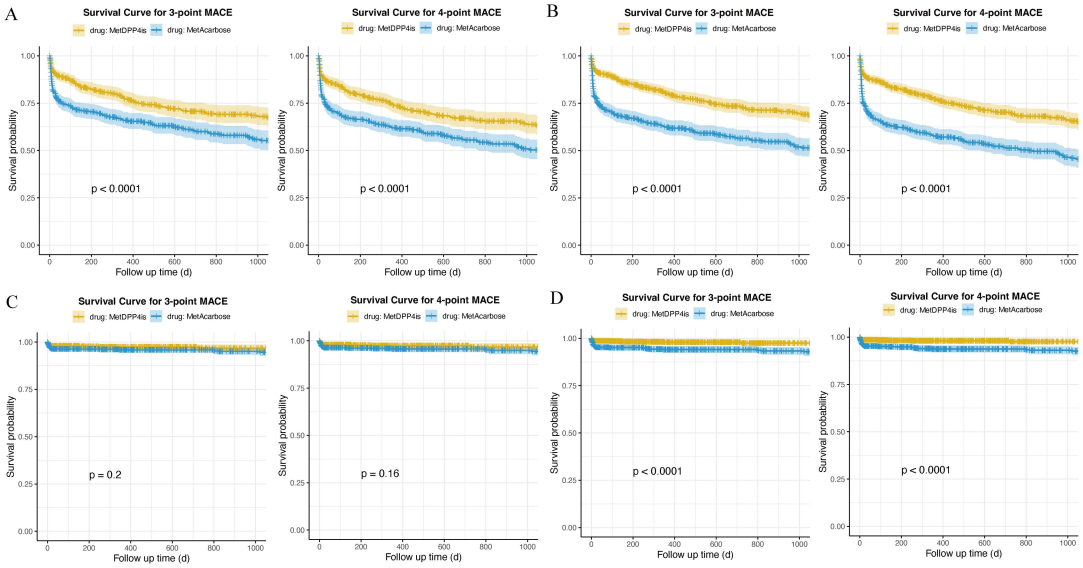

SFigure 4a: Survival curves for 3-point and 4-point MACE comparing MetAcarbose and MetDPP4is using PSM and IPTW methods in the JSPH and FAHZU Databases. Panel A represents results from the JSPH database using PSM, Panel B represents results from the JSPH database using IPTW, Panel C represents results from the FAHZU database using PSM, and Panel D represents results from the FAHZU database using IPTW. The x-axis indicates follow-up time (days), and the y-axis represents survival probability.

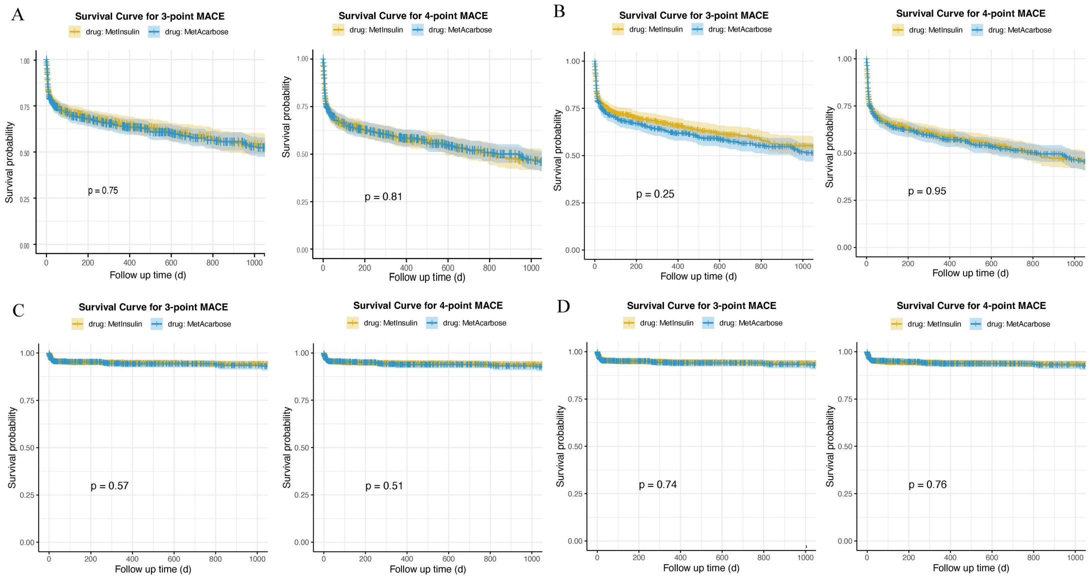

SFigure 4b: Survival curves for 3-point and 4-point MACE comparing MetAcarbose and MetInsulin using PSM and IPTW methods in the JSPH and FAHZU Databases. Panel A represents results from the JSPH database using PSM, Panel B represents results from the JSPH database using IPTW, Panel C represents results from the FAHZU database using PSM, and Panel D represents results from the FAHZU database using IPTW. The x-axis indicates follow-up time (days), and the y-axis represents survival probability.

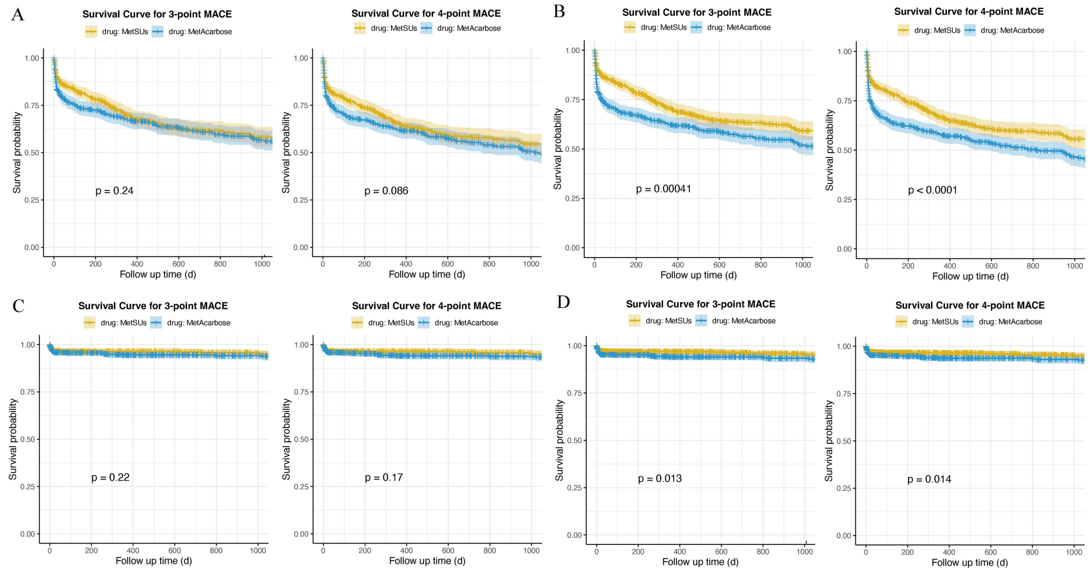

SFigure 4c: Survival curves for 3-point and 4-point MACE comparing MetAcarbose and MetSUs using PSM and IPTW methods in the JSPH and FAHZU Databases. Panel A represents results from the JSPH database using PSM, Panel B represents results from the JSPH database using IPTW, Panel C represents results from the FAHZU database using PSM, and Panel D represents results from the FAHZU database using IPTW. The x-axis indicates follow-up time (days), and the y-axis represents survival probability.

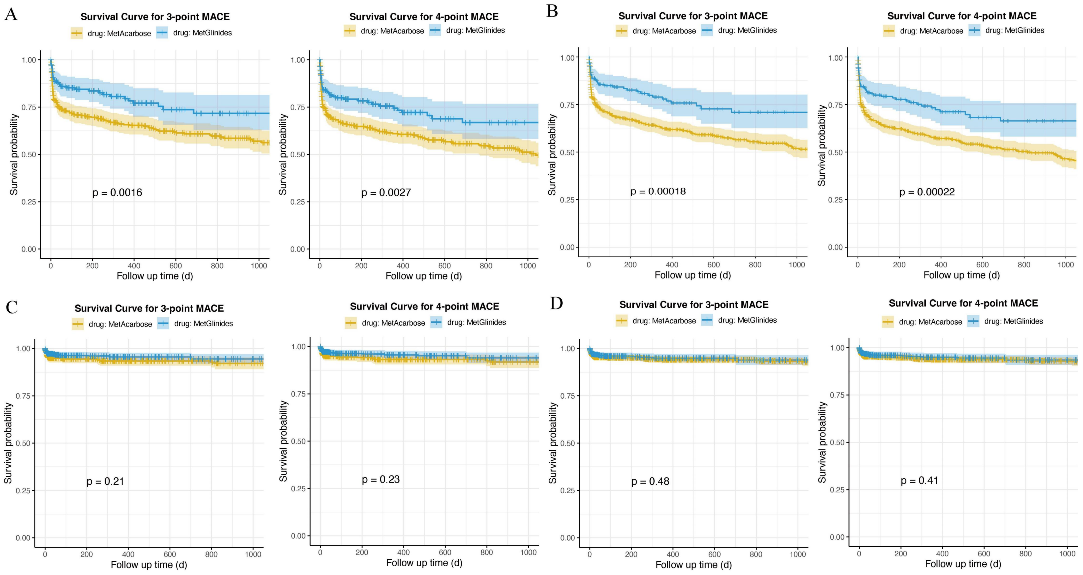

SFigure 4d: Survival curves for 3-point and 4-point MACE comparing MetGlinides and MetAcarbose using PSM and IPTW methods in the JSPH and FAHZU Databases. Panel A represents results from the JSPH database using PSM, Panel B represents results from the JSPH database using IPTW, Panel C represents results from the FAHZU database using PSM, and Panel D represents results from the FAHZU database using IPTW. The x-axis indicates follow-up time (days), and the y-axis represents survival probability.

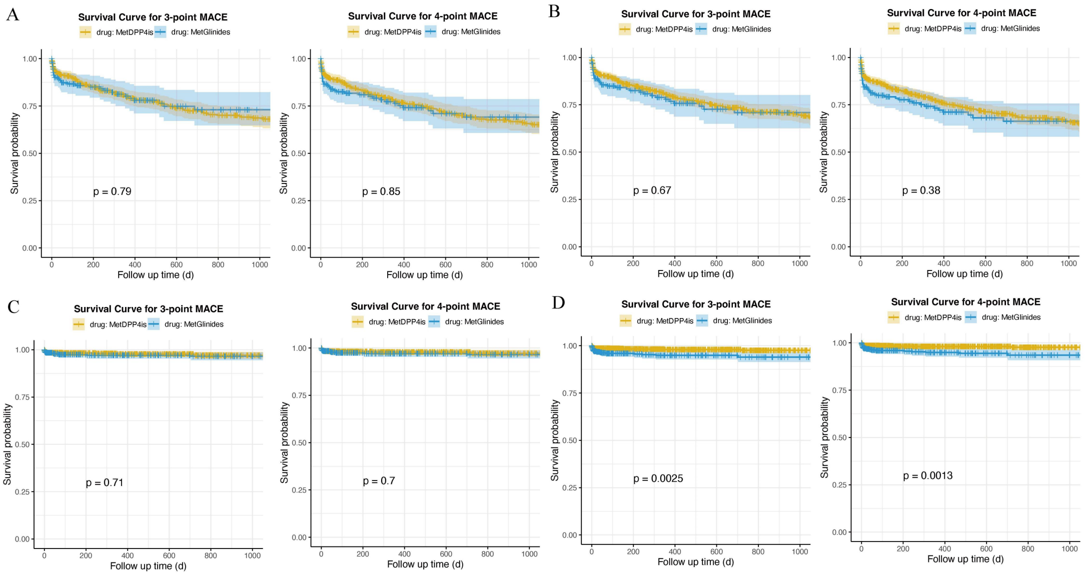

SFigure 4e: Survival curves for 3-point and 4-point MACE comparing MetGlinides and MetDPP4is using PSM and IPTW methods in the JSPH and FAHZU Databases. Panel A represents results from the JSPH database using PSM, Panel B represents results from the JSPH database using IPTW, Panel C represents results from the FAHZU database using PSM, and Panel D represents results from the FAHZU database using IPTW. The x-axis indicates follow-up time (days), and the y-axis represents survival probability.

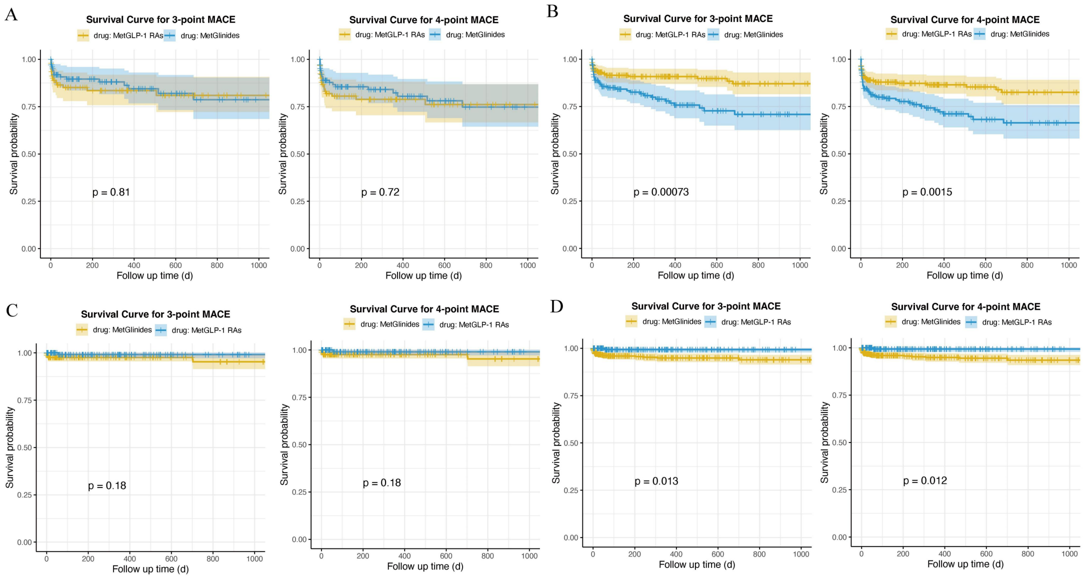

SFigure 4f: Survival curves for 3-point and 4-point MACE comparing MetGlinides and MetGLP-1 RAs using PSM and IPTW methods in the JSPH and FAHZU Databases. Panel A represents results from the JSPH database using PSM, Panel B represents results from the JSPH database using IPTW, Panel C represents results from the FAHZU database using PSM, and Panel D represents results from the FAHZU database using IPTW. The x-axis indicates follow-up time (days), and the y-axis represents survival probability.

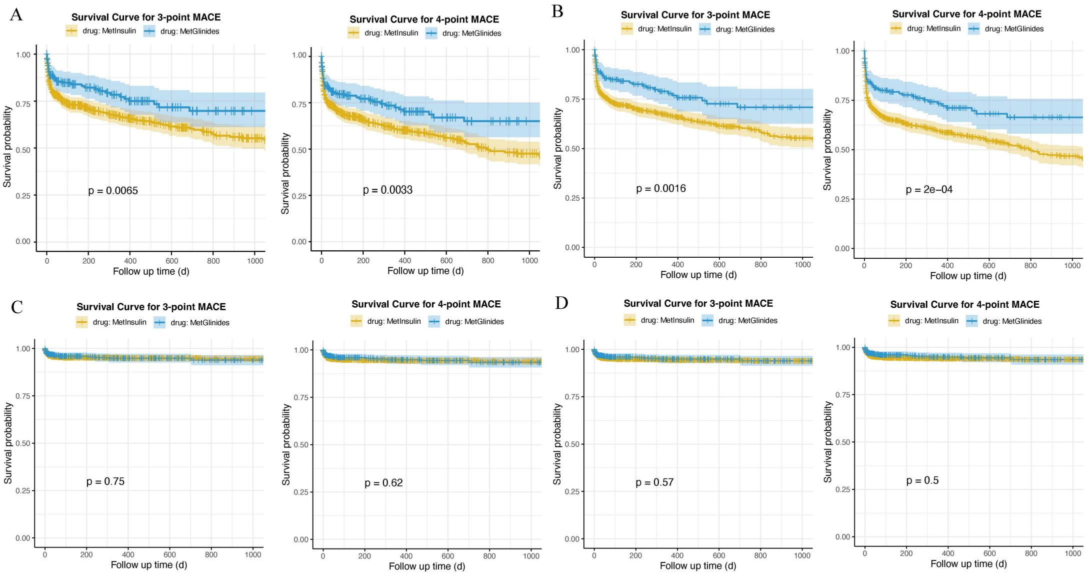

SFigure 4g: Survival curves for 3-point and 4-point MACE comparing MetGlinides and MetInsulin using PSM and IPTW methods in the JSPH and FAHZU Databases. Panel A represents results from the JSPH database using PSM, Panel B represents results from the JSPH database using IPTW, Panel C represents results from the FAHZU database using PSM, and Panel D represents results from the FAHZU database using IPTW. The x-axis indicates follow-up time (days), and the y-axis represents survival probability.

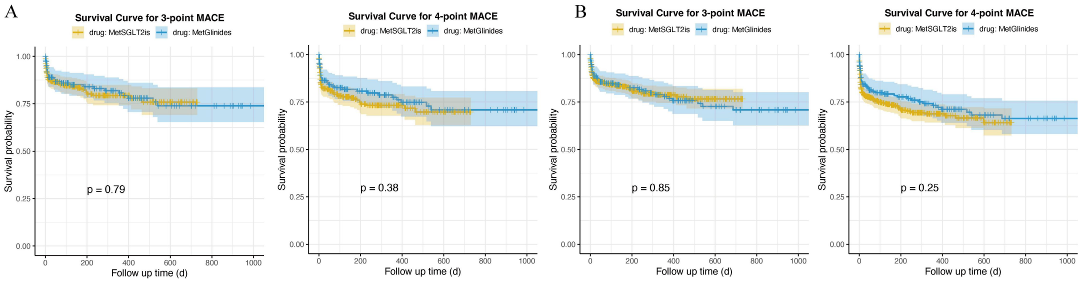

SFigure 4h: Survival curves for 3-point and 4-point MACE comparing MetGlinides and MetSGLT2is using PSM and IPTW methods in the JSPH and FAHZU Databases. Panel A represents results from the JSPH database using PSM, and Panel B represents results from the JSPH database using IPTW. Due to data unavailability, Panels C and D for the FAHZU database using PSM and IPTW are not presented. The x-axis indicates follow-up time (days), and the y-axis represents survival probability.

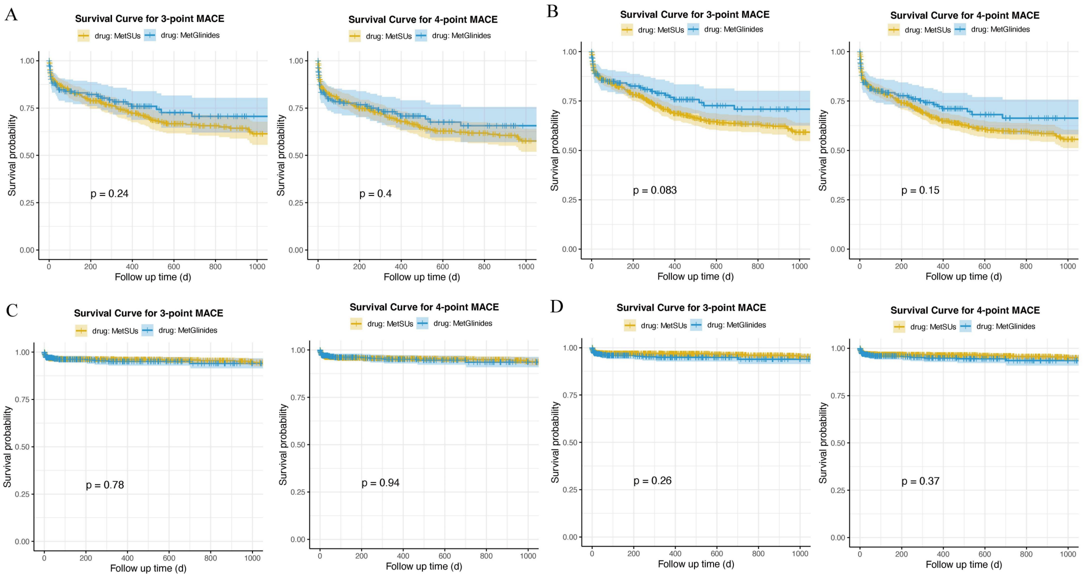

SFigure 4i: Survival curves for 3-point and 4-point MACE comparing MetGlinides and MetSUs using PSM and IPTW methods in the JSPH and FAHZU Databases. Panel A represents results from the JSPH database using PSM, Panel B represents results from the JSPH database using IPTW, Panel C represents results from the FAHZU database using PSM, and Panel D represents results from the FAHZU database using IPTW. The x-axis indicates follow-up time (days), and the y-axis represents survival probability.

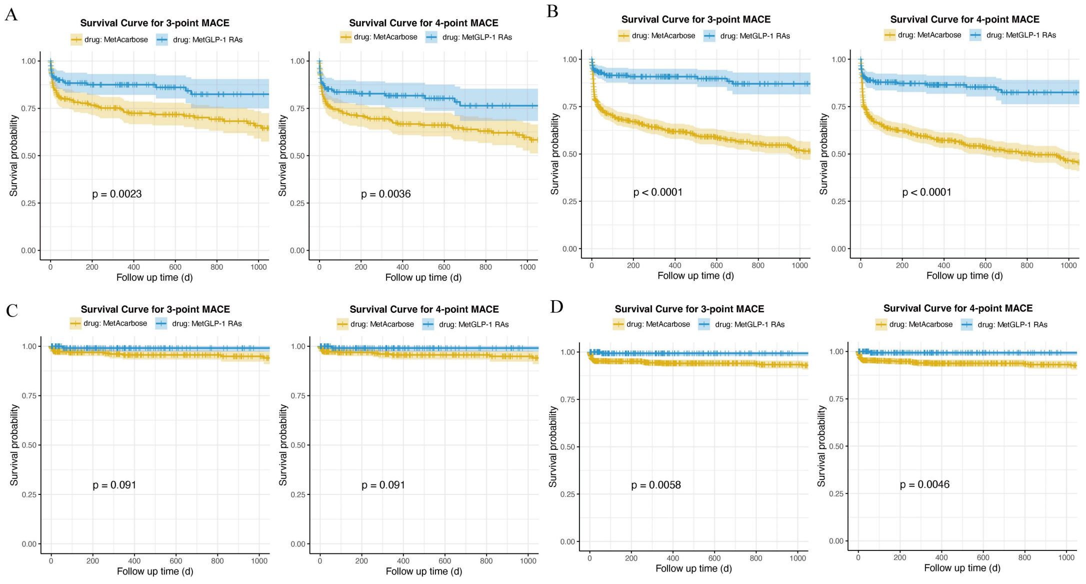

SFigure 4j: Survival curves for 3-point and 4-point MACE comparing MetGLP-1 RAs and MetAcarbose using PSM and IPTW methods in the JSPH and FAHZU Databases. Panel A represents results from the JSPH database using PSM, Panel B represents results from the JSPH database using IPTW, Panel C represents results from the FAHZU database using PSM, and Panel D represents results from the FAHZU database using IPTW. The x-axis indicates follow-up time (days), and the y-axis represents survival probability.

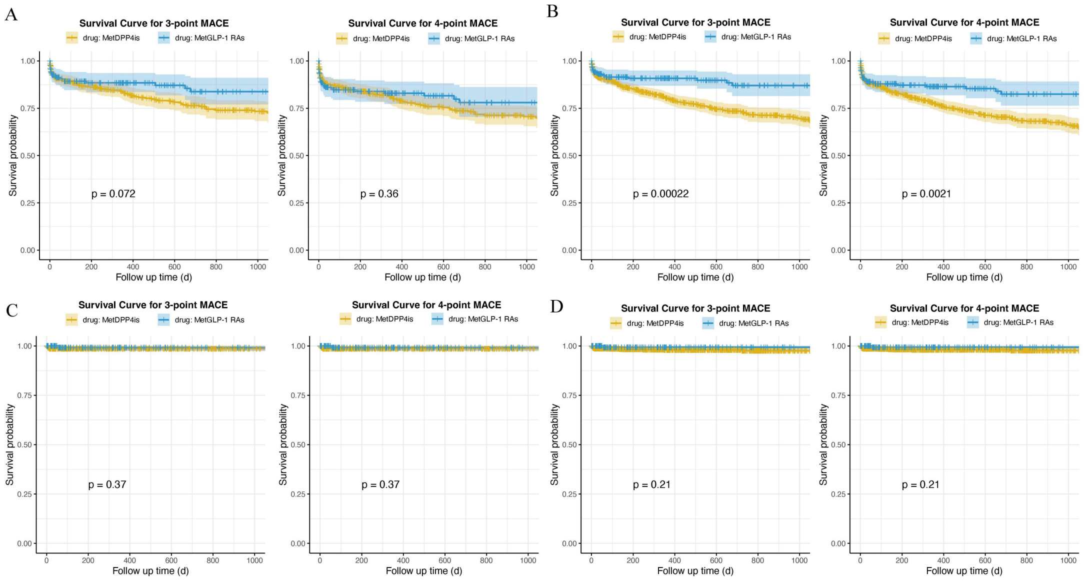

SFigure 4k: Survival curves for 3-point and 4-point MACE comparing MetGLP-1 RAs and MetDPP4is using PSM and IPTW methods in the JSPH and FAHZU Databases. Panel A represents results from the JSPH database using PSM, Panel B represents results from the JSPH database using IPTW, Panel C represents results from the FAHZU database using PSM, and Panel D represents results from the FAHZU database using IPTW. The x-axis indicates follow-up time (days), and the y-axis represents survival probability.

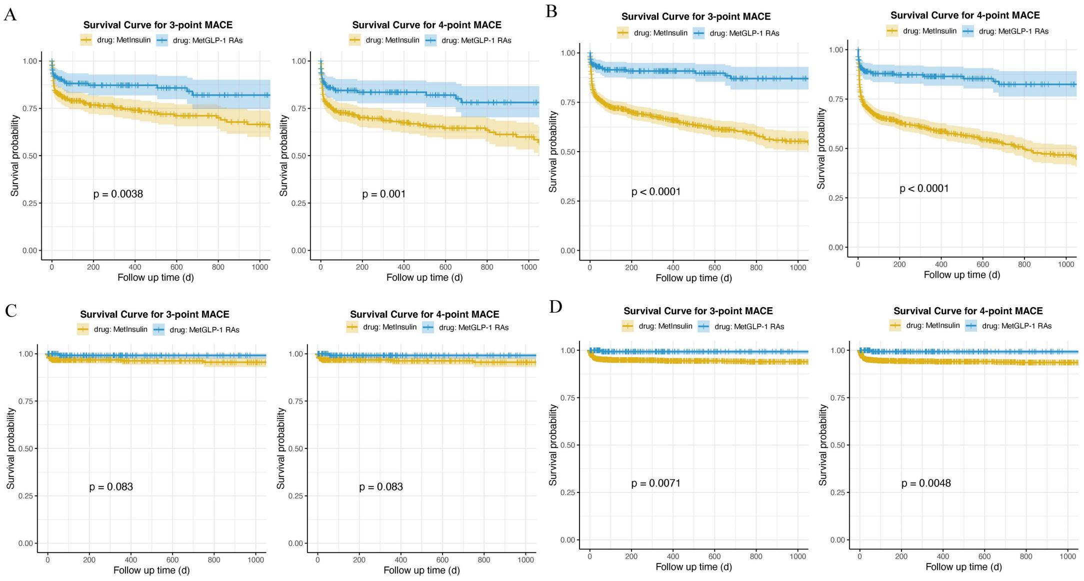

SFigure 4l: Survival curves for 3-point and 4-point MACE comparing MetGLP-1 RAs and MetInsulin using PSM and IPTW methods in the JSPH and FAHZU Databases. Panel A represents results from the JSPH database using PSM, Panel B represents results from the JSPH database using IPTW, Panel C represents results from the FAHZU database using PSM, and Panel D represents results from the FAHZU database using IPTW. The x-axis indicates follow-up time (days), and the y-axis represents survival probability.

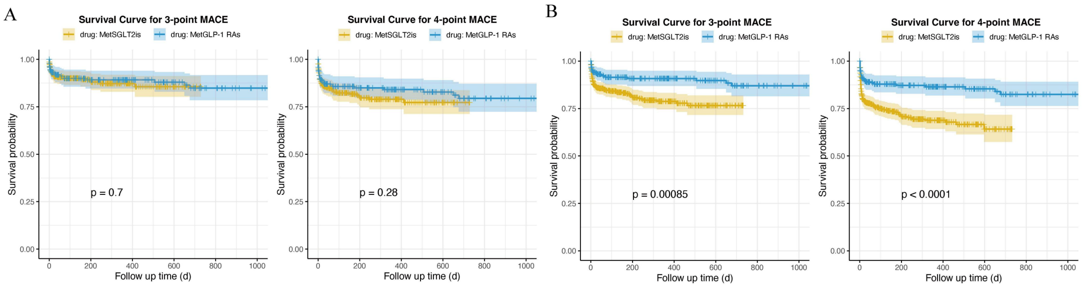

SFigure 4m: Survival curves for 3-point and 4-point MACE comparing MetGLP-1 RAs and MetSGLT2is using PSM and IPTW methods in the JSPH and FAHZU Databases. Panel A represents results from the JSPH database using PSM, and Panel B represents results from the JSPH database using IPTW. Due to data unavailability, Panels C and D for the FAHZU database using PSM and IPTW are not presented. The x-axis indicates follow-up time (days), and the y-axis represents survival probability.

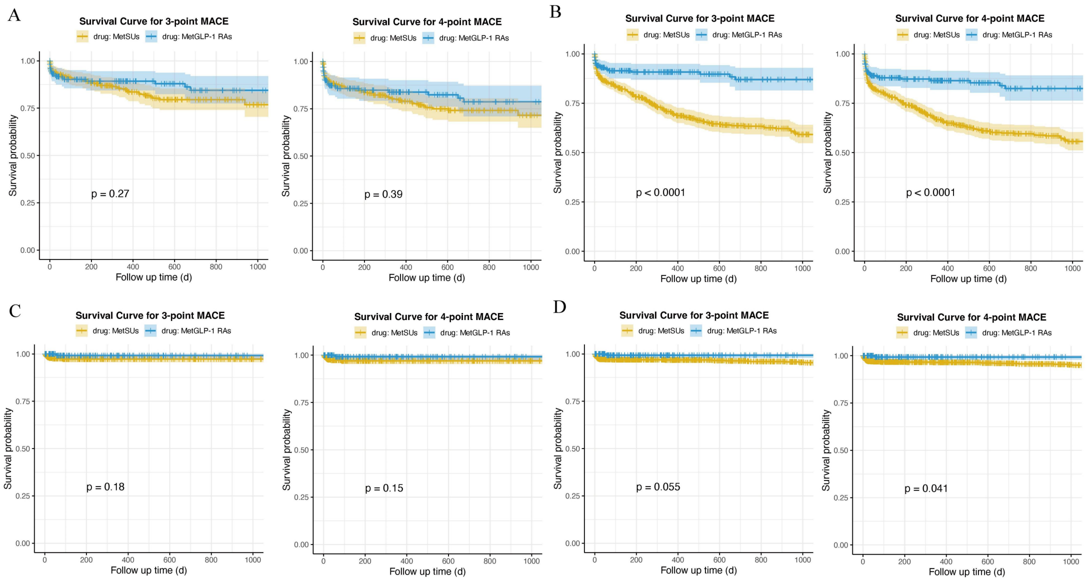

SFigure 4n: Survival curves for 3-point and 4-point MACE comparing MetGLP-1 RAs and MetSUs using PSM and IPTW methods in the JSPH and FAHZU Databases. Panel A represents results from the JSPH database using PSM, Panel B represents results from the JSPH database using IPTW, Panel C represents results from the FAHZU database using PSM, and Panel D represents results from the FAHZU database using IPTW. The x-axis indicates follow-up time (days), and the y-axis represents survival probability.

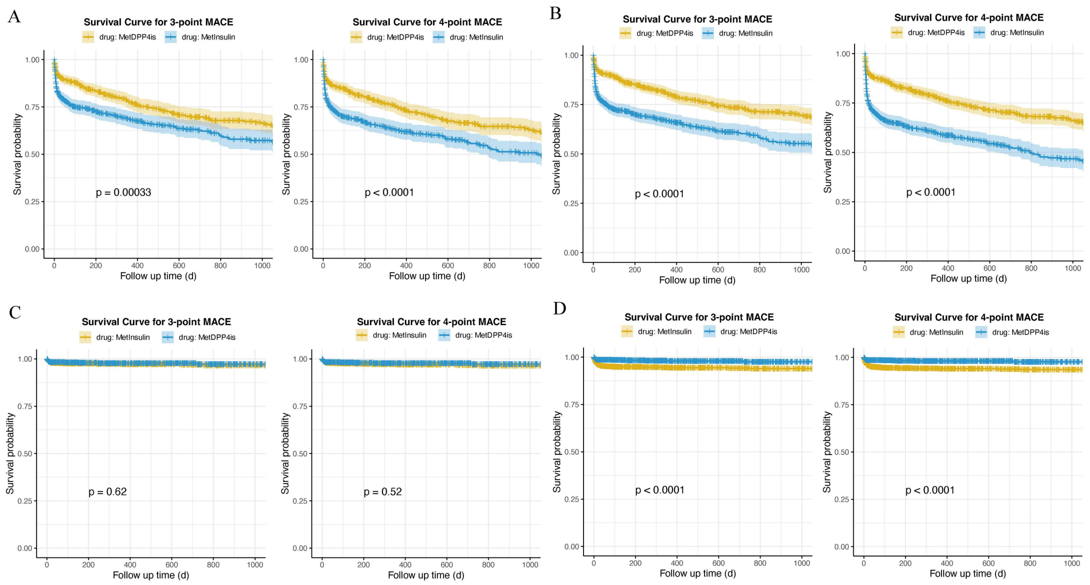

SFigure 4o: Survival curves for 3-point and 4-point MACE comparing MetInsulin and MetDPP4is using PSM and IPTW methods in the JSPH and FAHZU Databases. Panel A represents results from the JSPH database using PSM, Panel B represents results from the JSPH database using IPTW, Panel C represents results from the FAHZU database using PSM, and Panel D represents results from the FAHZU database using IPTW. The x-axis indicates follow-up time (days), and the y-axis represents survival probability.

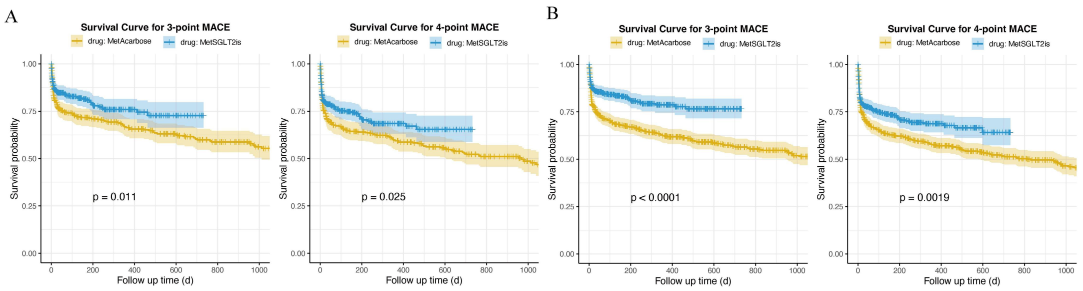

SFigure 4p: Survival curves for 3-point and 4-point MACE comparing MetSGLT2is and MetAcarbose using PSM and IPTW methods in the JSPH and FAHZU Databases. Panel A represents results from the JSPH database using PSM, and Panel B represents results from the JSPH database using IPTW. Due to data unavailability, Panels C and D for the FAHZU database using PSM and IPTW are not presented. The x-axis indicates follow-up time (days), and the y-axis represents survival probability.

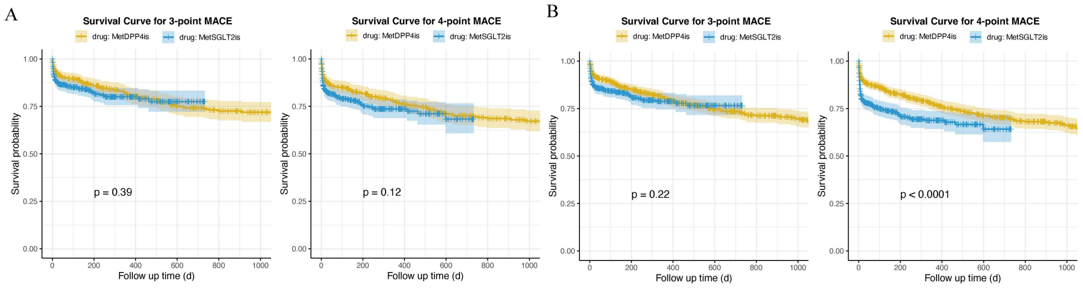

SFigure 4q: Survival curves for 3-point and 4-point MACE comparing MetSGLT2is and MetDPP4is using PSM and IPTW methods in the JSPH and FAHZU Databases. Panel A represents results from the JSPH database using PSM, and Panel B represents results from the JSPH database using IPTW. Due to data unavailability, Panels C and D for the FAHZU database using PSM and IPTW are not presented. The x-axis indicates follow-up time (days), and the y-axis represents survival probability.

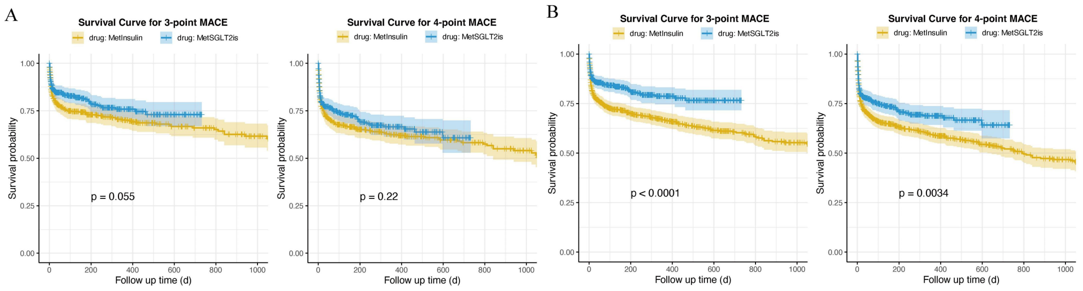

SFigure 4r: Survival curves for 3-point and 4-point MACE comparing MetSGLT2is and MetInsulin using PSM and IPTW methods in the JSPH and FAHZU Databases. Panel A represents results from the JSPH database using PSM, and Panel B represents results from the JSPH database using IPTW. Due to data unavailability, Panels C and D for the FAHZU database using PSM and IPTW are not presented. The x-axis indicates follow-up time (days), and the y-axis represents

survival probability.

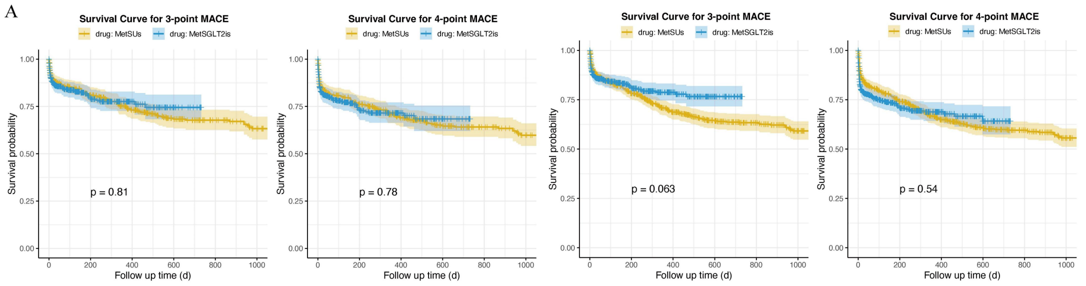

SFigure 4s: Survival curves for 3-point and 4-point MACE comparing MetSGLT2is and MetSUs using PSM and IPTW methods in the JSPH and FAHZU Databases. Panel A represents results from the JSPH database using PSM, and Panel B represents results from the JSPH database using IPTW. Due to data unavailability, Panels C and D for the FAHZU database using PSM and IPTW are not presented. The x-axis indicates follow-up time (days), and the y-axis represents survival probability.

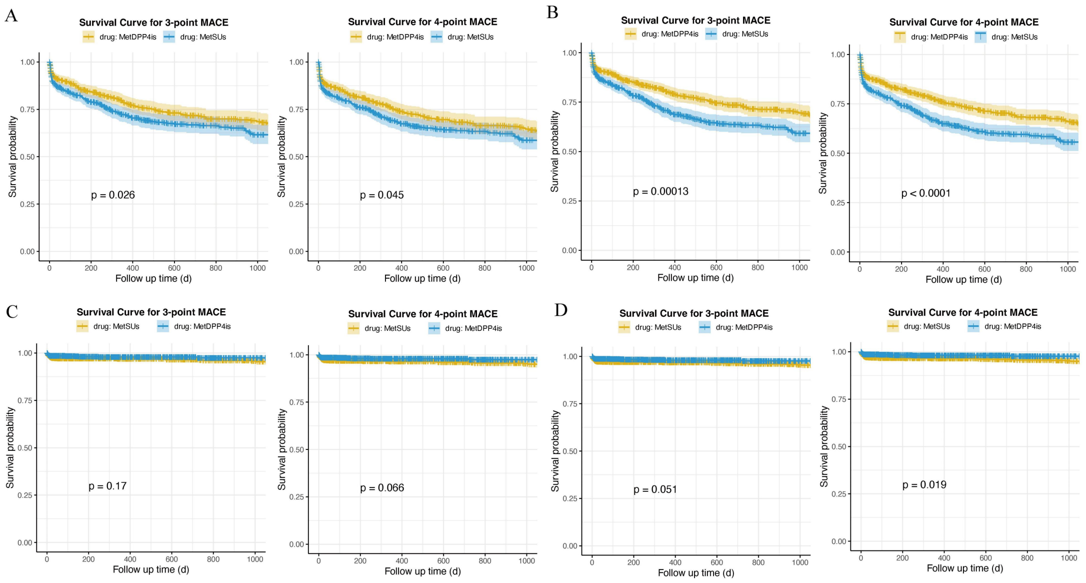

SFigure 4t: Survival curves for 3-point and 4-point MACE comparing MetSUs and MetDPP4is using PSM and IPTW methods in the JSPH and FAHZU Databases. Panel A represents results from the JSPH database using PSM, Panel B represents results from the JSPH database using IPTW, Panel C represents results from the FAHZU database using PSM, and Panel D represents results from the FAHZU database using IPTW. The x-axis indicates follow-up time (days), and the y-axis represents survival probability.

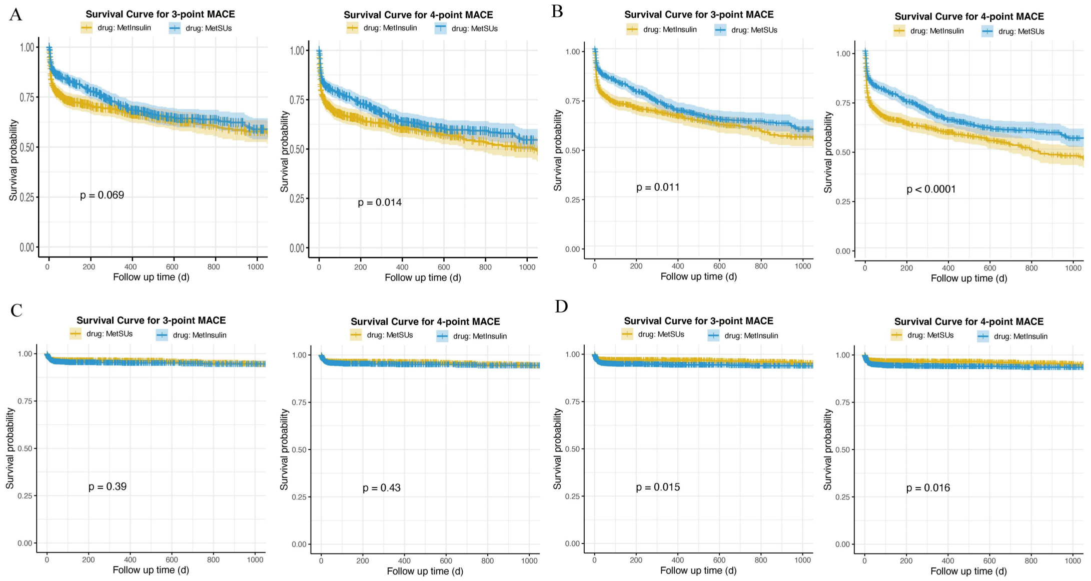

SFigure 4u: Survival curves for 3-point and 4-point MACE comparing MetSUs and MetInsulin using PSM and IPTW methods in the JSPH and FAHZU Databases. Panel A represents results from the JSPH database using PSM, Panel B represents results from the JSPH database using IPTW, Panel C represents results from the FAHZU database using PSM, and Panel D represents results from the FAHZU database using IPTW. The x-axis indicates follow-up time (days), and the y-axis represents survival probability.

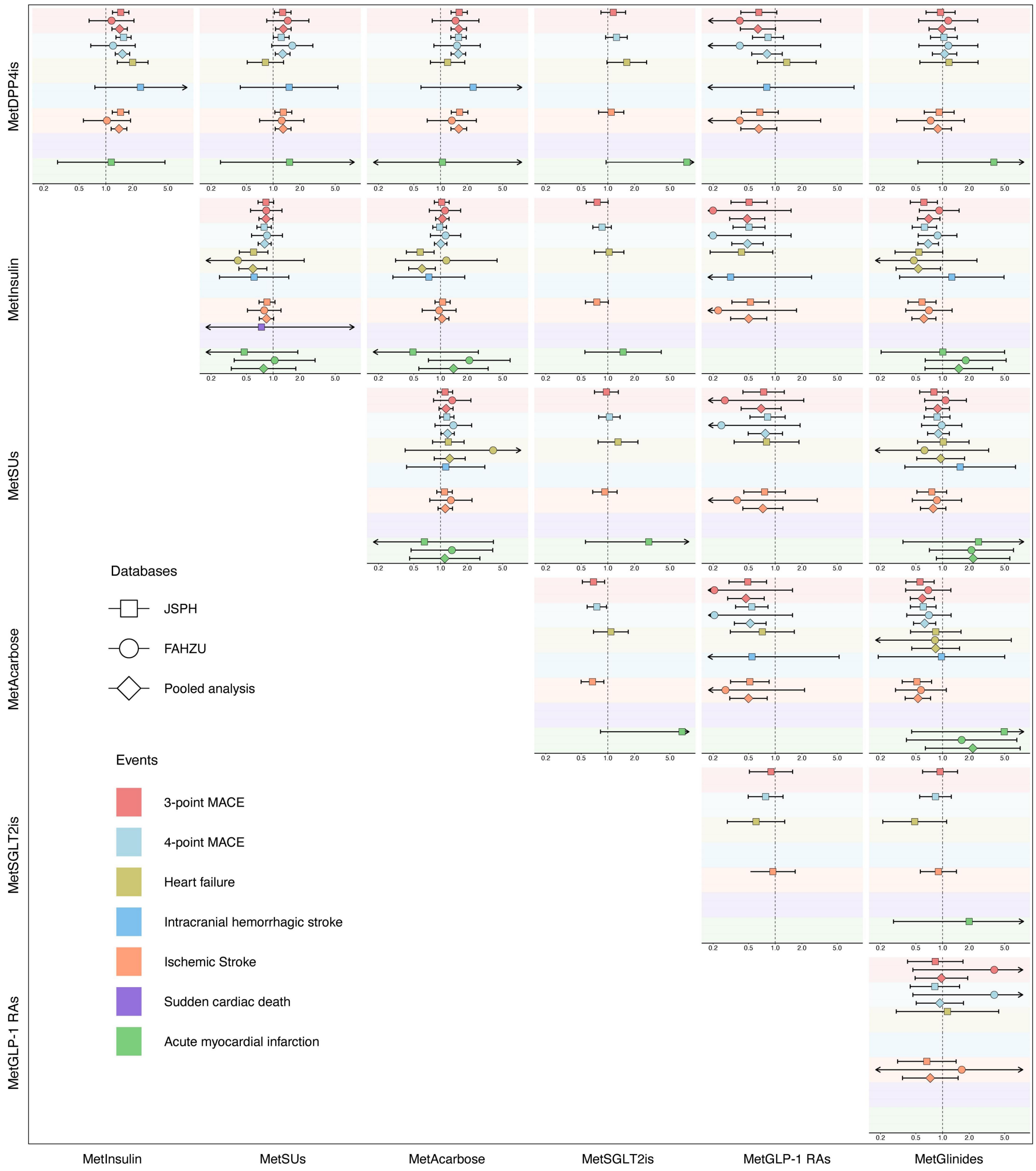

SFigure 5: Comparative effectiveness of hypoglycemic drug classes on seven cardiovascular events using PSM method in JSPH, FAHZU, and Pooled analysis. For each drug comparison, the target drug class is represented in the row, while the comparator drug class is represented in the column. Points report HR estimates, with lines marking their 95% CIs. An HR > 1 indicates that the risk is higher in the target drug class, while an HR < 1 indicates that it is higher in the comparator drug class. Comparisons were considered statistically significant if the 95% CI did not overlap 1.

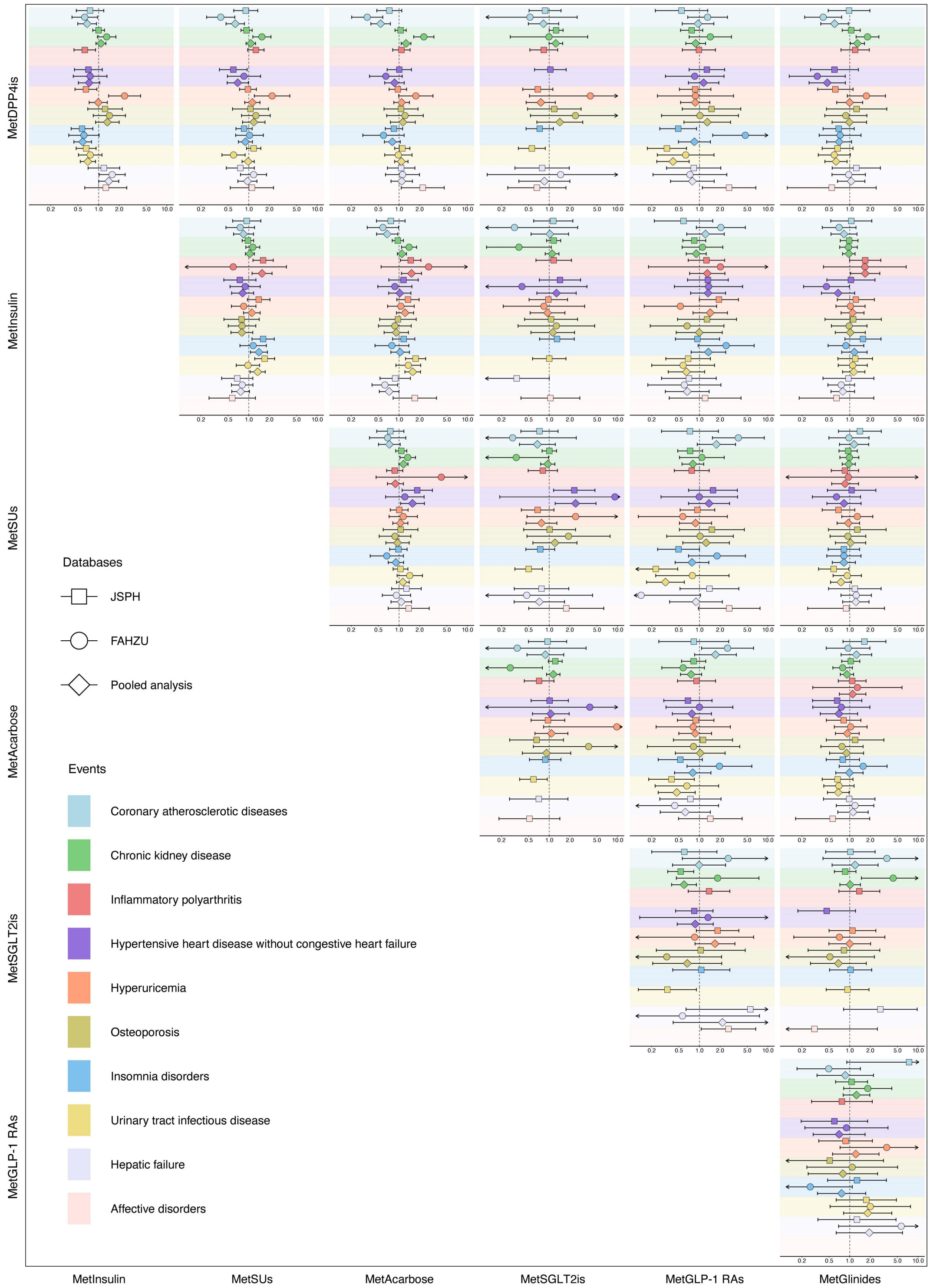

SFigure 6: Comparative safety profiles of hypoglycemic drug classes on ten events using PSM method in JSPH, FAHZU, and Pooled analysis. For each drug comparison, the target drug class is represented in the row, while the comparator drug class is represented in the column. Points report HR estimates, with lines marking their 95% CIs. An HR > 1 indicates that the risk is higher in the target drug class, while an HR < 1 indicates that it is higher in the comparator drug class. Comparisons were considered statistically significant if the 95% CI did not overlap 1.

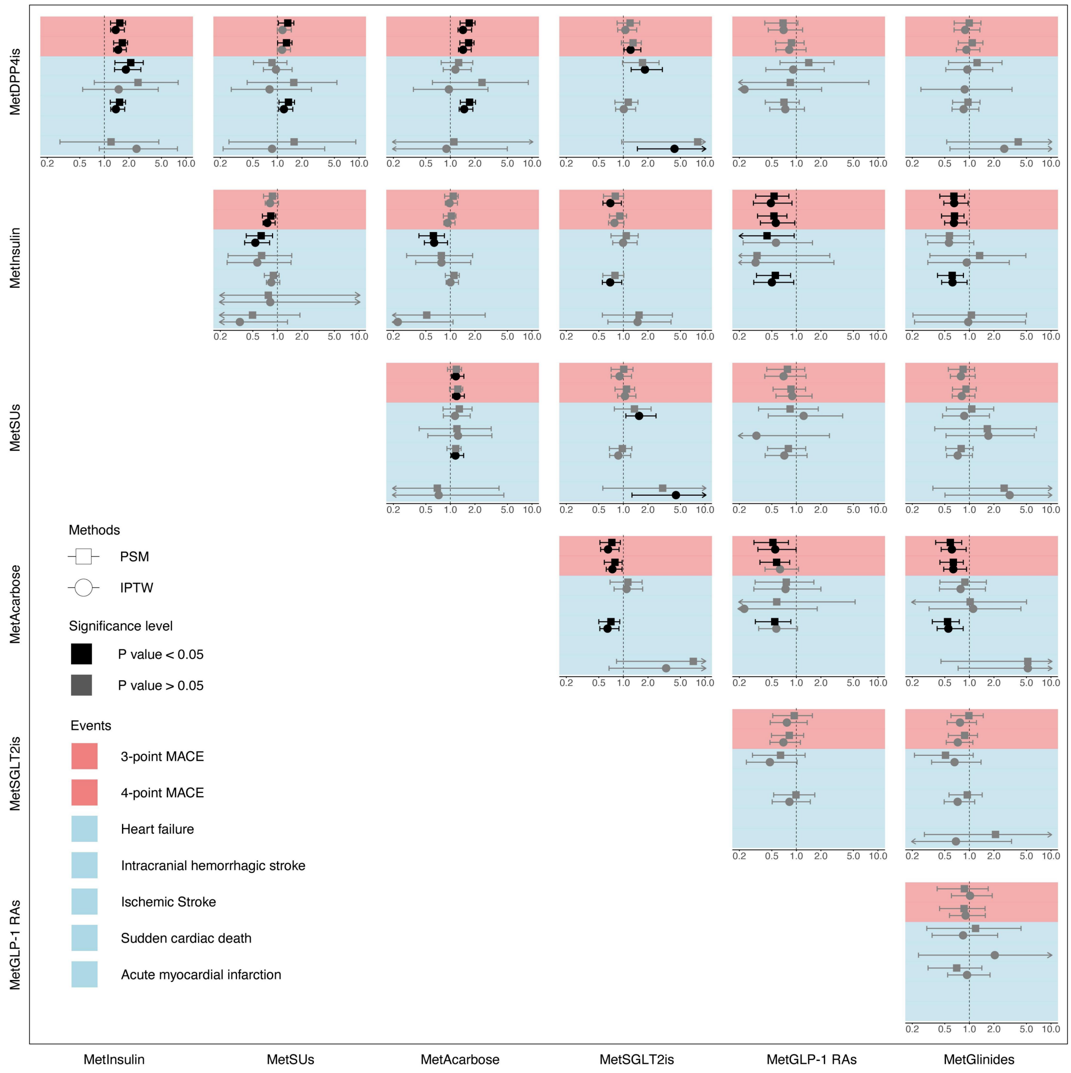

SFigure 7a: Sensitivity analysis of hypoglycemic drug classes on seven cardiovascular events using PSM and IPTW methods in the JSPH database. For each drug comparison, the target drug class is in the row, while the comparator drug class is in the column. Points report HR estimates, with lines marking their 95% CIs. Black points represent statistically significant comparisons (*P value* < 0.05), while gray points represent non-significant comparisons *(P value* > 0.05). An HR > 1 indicates that the risk is higher in the target drug class, while an HR < 1 indicates that it is higher in the comparator drug class.

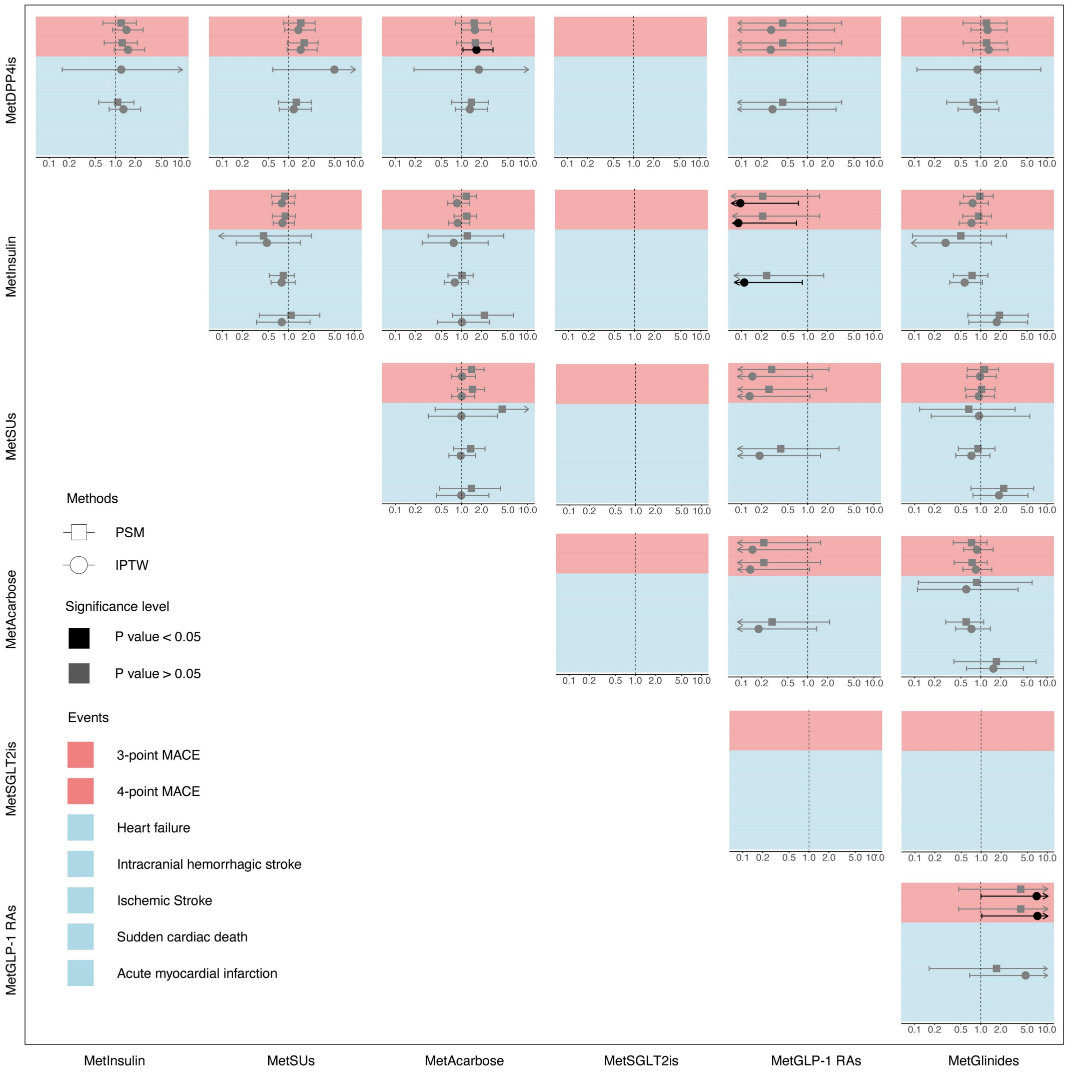

SFigure 7b: Sensitivity analysis of hypoglycemic drug classes on seven cardiovascular events using PSM and IPTW methods in the FAHZU database. For each drug comparison, the target drug class is in the row, while the comparator drug class is in the column. Points report HR estimates, with lines marking their 95% CIs. Black points represent statistically significant comparisons (*P value* < 0.05), while gray points represent non-significant comparisons (*P value* > 0.05). An HR > 1 indicates that the risk is higher in the target drug class, while an HR < 1 indicates that it is higher in the comparator drug class.

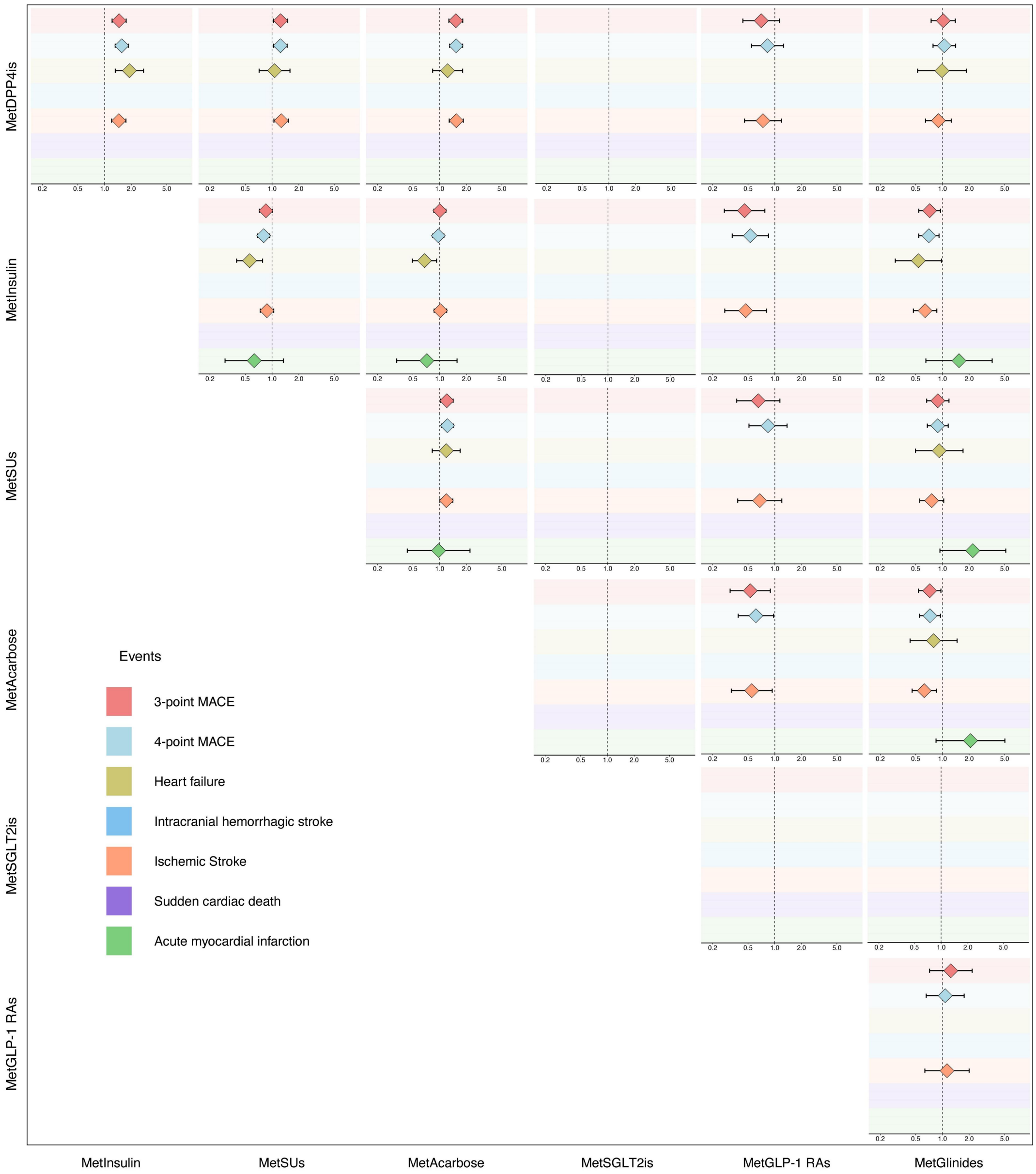

SFigure 7c: Sensitivity analysis of hypoglycemic drug classes on seven cardiovascular events using IPTW methods in the Pooled analysis. For each drug comparison, the target drug class is represented in the row, while the comparator drug class is represented in the column. Points report HR estimates, with lines marking their 95% CIs. An HR > 1 indicates that the risk is higher in the target drug class, while an HR < 1 indicates that it is higher in the comparator drug class. Comparisons were considered statistically significant if the 95% CI did not overlap 1.

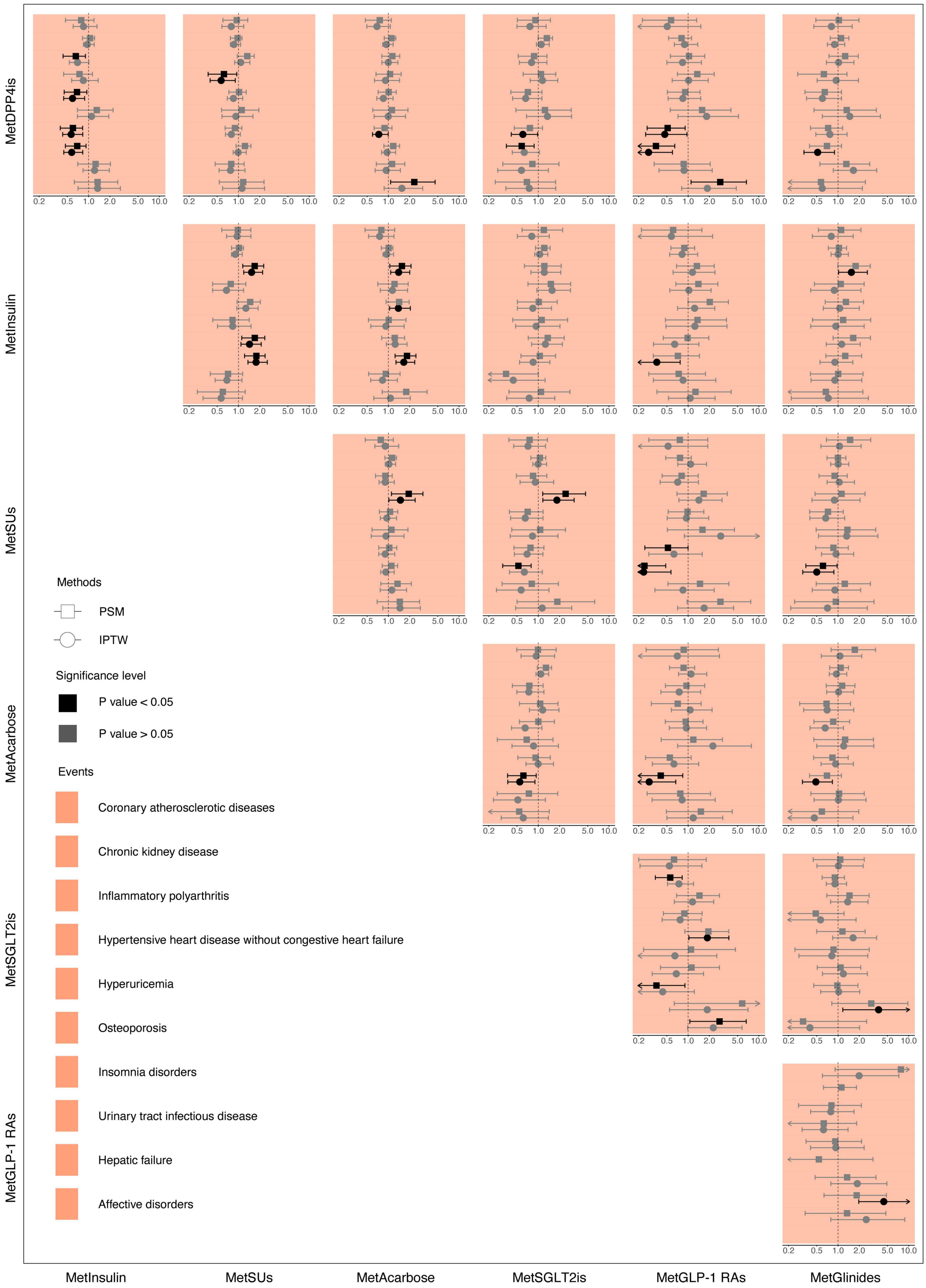

SFigure 8a: Sensitivity analysis of hypoglycemic drug classes on ten events using PSM and IPTW methods in the JSPH database. For each drug comparison, the target drug class is in the row, while the comparator drug class is in the column. Points report HR estimates, with lines marking their 95% CIs. Black points represent statistically significant comparisons (*P value* < 0.05), while gray points represent non-significant comparisons (*P value* > 0.05). An HR > 1 indicates that the risk is higher in the target drug class, while an HR < 1 indicates that it is higher in the comparator drug class.

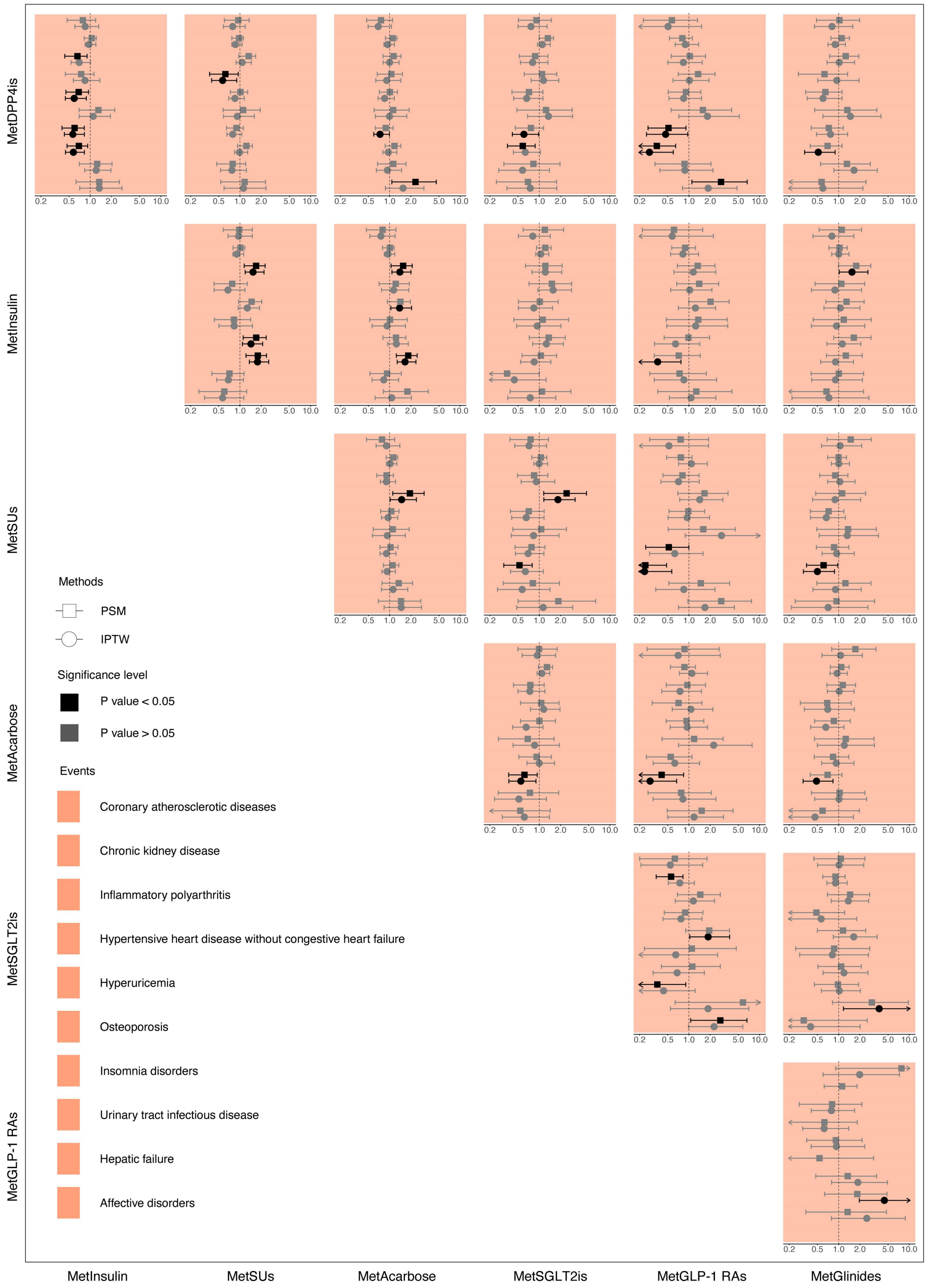

SFigure 8b: Sensitivity analysis of hypoglycemic drug classes on ten events using PSM and IPTW methods in the FAHZU database. For each drug comparison, the target drug class is in the row, while the comparator drug class is in the column. Points report HR estimates, with lines marking their 95% CIs. Black points represent statistically significant comparisons (*P value* < 0.05), while gray points represent non-significant comparisons (*P value* > 0.05). An HR > 1 indicates that the risk is higher in the target drug class, while an HR < 1 indicates that it is higher in the comparator drug class.

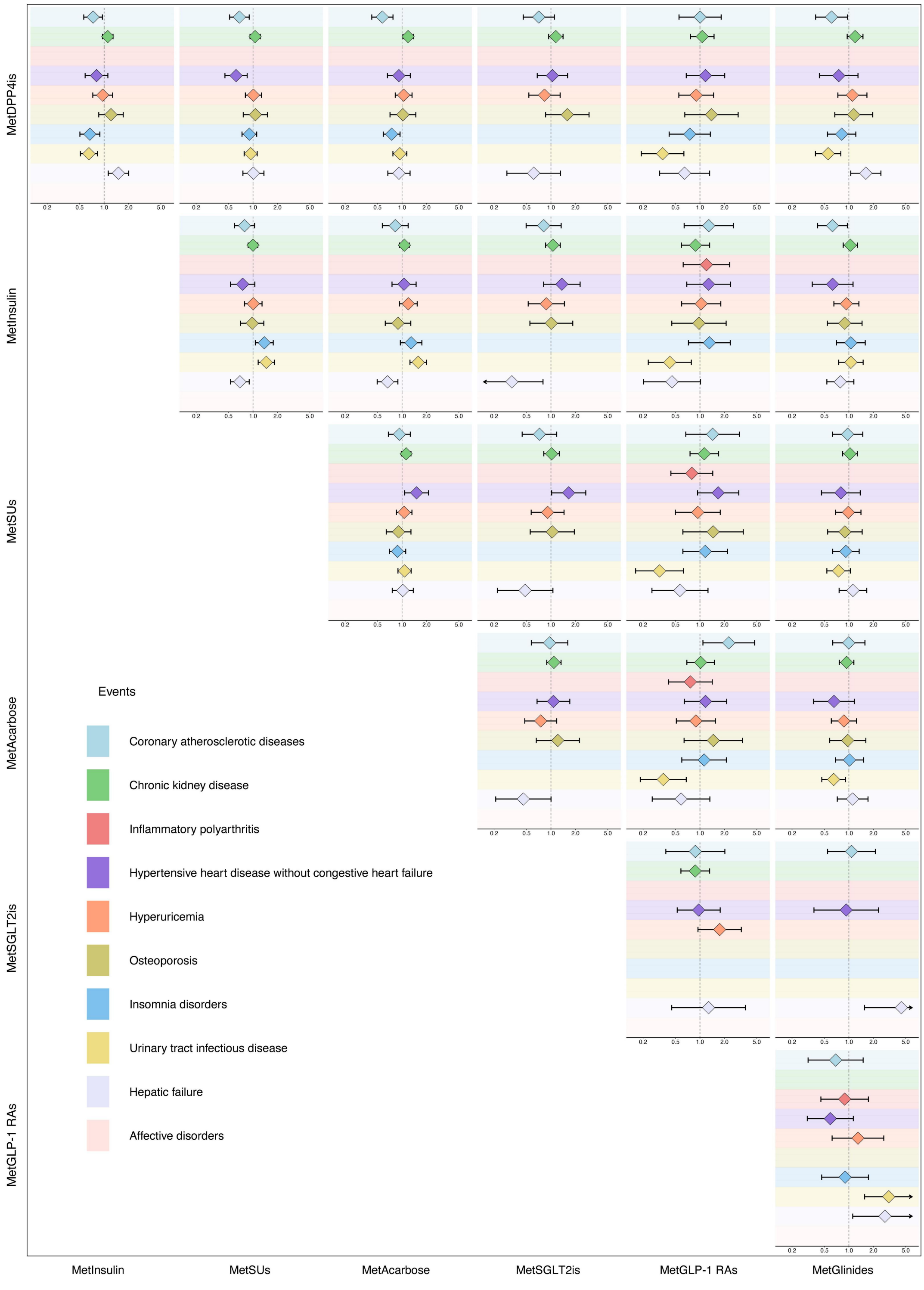

SFigure 8c: Sensitivity analysis of hypoglycemic drug classes on ten events using IPTW methods in the Pooled analysis. For each drug comparison, the target drug class is represented in the row, while the comparator drug class is represented in the column. Points report HR estimates, with lines marking their 95% CIs. An HR > 1 indicates that the risk is higher in the target drug class, while an HR < 1 indicates that it is higher in the comparator drug class. Comparisons were considered statistically significant if the 95% CI did not overlap 1.
